# Mapping of Family Reported Outcome Measure (FROM-16) scores to EQ-5D: algorithm to calculate utility values

**DOI:** 10.1007/s11136-023-03590-z

**Published:** 2024-02-25

**Authors:** R. Shah, M. S. Salek, A. Y. Finlay, R. Kay, S. J. Nixon, K. Otwombe, F. M. Ali, J. R. Ingram

**Affiliations:** 1 Division of Infection and Immunity, School of Medicine, Cardiff University, Cardiff, UK; 2 School of Life & Medical Sciences, University of Hertfordshire, Hatfield, UK; 3 RK Statistics, Bakewell, UK; 4 Multiple Sclerosis Society, Cardiff, UK; 5 Statistics and Data Management Centre, Perinatal HIV Research Unit, University of the Witwatersrand, Johannesburg, South Africa

**Keywords:** FROM-16, EQ-5D, Utility values, Response mapping, Health technology, Health economic evaluation, Family member/partner, Informal carer, Quality of life

## Abstract

**Objective:**

Although decision scientists and health economists encourage inclusion of family member/informal carer utility in health economic evaluation, there is a lack of suitable utility measures comparable to patient utility measures such those based on the EQ-5D. This study aims to predict EQ-5D-3L utility values from Family Reported Outcome Measure (FROM-16) scores, to allow the use of FROM-16 data in health economic evaluation when EQ-5D data is not available.

**Methods:**

Data from 4228 family members/partners of patients recruited to an online cross-sectional study through 58 UK-based patient support groups, three research support platforms and Welsh social services departments were randomly divided five times into two groups, to derive and test a mapping model. Split-half cross-validation was employed, resulting in a total of ten multinomial logistic regression models. The Monte Carlo simulation procedure was used to generate predicted EQ-5D-3L responses, and utility scores were calculated and compared against observed values. Mean error and mean absolute error were calculated for all ten validation models. The final model algorithm was derived using the entire sample.

**Results:**

The model was highly predictive, and its repeated fitting using multinomial logistic regression demonstrated a stable model. The mean differences between predicted and observed health utility estimates ranged from 0.005 to 0.029 across the ten modelling exercises, with an average overall difference of 0.015 (a 2.2% overestimate, not of clinical importance).

**Conclusions:**

The algorithm developed will enable researchers and decision scientists to calculate EQ-5D health utility estimates from FROM-16 scores, thus allowing the inclusion of the family impact of disease in health economic evaluation of medical interventions when EQ-5D data is not available.

**Supplementary Information:**

The online version contains supplementary material available at 10.1007/s11136-023-03590-z.

## Introduction

Public healthcare decision-making is increasingly supported by economic analyses of healthcare interventions. Decision scientists and economists compare the costs and outcomes of novel interventions with the best alternative to inform their cost-effectiveness. Currently, such analysis is focused only on patient outcomes [1]. However, new treatments that improve patients’ quality of life (QoL) can also improve the QoL of their family members. Caring for one’s relative may leave family members/partners physically and emotionally drained. This impact is particularly high where there is a significant amount of caring, such as for a chronic neurological condition. Ignoring the potentially large impacts on QoL of family members/partners may result in inequitable and inaccurate evaluation of the medical intervention [2]. Although the inclusion of family member burden in economic evaluations is encouraged by many health technology assessment agencies, such as the National Institute for Health and Care Excellence (NICE), it is seldom reported. Some researchers attribute this to uncertainty about decision-makers’ attitudes toward their inclusion, issues over how the burden may be incorporated into economic models, and the availability of suitable utility measures for carers/family members [3]. The lack of carer data may be the most plausible explanation as to why this impact is not included in CEAs [1, 4, 5], as family member/informal carer inclusion in HTA is a recent recommendation with currently no family members/carer data being collected in clinical trials or alongside patient registries.

In the UK, NICE uses the Quality Adjusted Life Year (QALY), a composite measure of quality and quantity of life, to quantify the health effect of a medical intervention and ultimately inform resource allocation. In order to generate QALYs, health utilities (or HRQoL weights) are needed, and the NICE preferred measure is the European Quality of Life-5 Dimensions three-Level (EQ-5D-3L) [6]. Some authors argue that generic preference-based measures (PBMs) such as EQ-5D may not be adequate to assess carer utility as they were not designed for this purpose [7]. Although the CarerQoL [8] and Carer Experience Scale (CES) [9] have been valued using choice-based methods, these cannot be used to estimate utility weights [7]. Nevertheless, generic PBMs have been used successfully to assess family member/informal carer utilities, with EQ-5D being the most common generic instrument to measure Carer utility [10]. Evidence from a recent study comparing five QoL instruments for carers across four conditions has shown that EQ-5D had some validity and may be appropriate for use in health technology evaluations [11, 12]. The main advantage of using the EQ-5D to measure family member/informal carer QoL is that it can easily be combined with patient QoL, allowing greater comparability across appraisals. Therefore, mapping family-specific QoL measures such as the Family Reported Outcome Measure (FROM-16) to EQ-5D will allow the inclusion of family members and/or informal carers in health economic evaluation when EQ-5D data is not available. The FROM-16 measures QoL impact of a patient’s disease on their family members/partners across all areas of medicine [13]. It is validated and translated into many languages [14–17]: score descriptor bands have been calculated [18]. Mapping FROM-16 to EQ-5D would enable the calculation of QALYs for family members and/or informal carers allowing comparability with patient utilities.

Direct mapping uses either the total or subdomain scores to predict Preference-based measure utility values, while response mapping predicts EQ-5D responses for utilities from the responses on other measures. The most common approach used for direct mapping is the Ordinary Least Square (OLS), that has several limitations. First, it suggests that utilities are continuously distributed and therefore, the utility value of 1.0 cannot be achieved [19]. Secondly, in the case of ceiling effects, OLS can produce inconsistent estimates of the coefficients of explanatory variables. Although other methods of direct mapping have been explored to overcome these issues [20, 21], these methods can only provide mapping for a single set of utility values relevant to the country of tariff. In contrast, response mapping predicts EQ-5D dimension responses, which can be used to derive utility values using any country-specific tariff [19].

In this study, we use response mapping to predict EQ-5D health utility estimates from FROM-16 responses to allow the use of FROM-16 in health economic evaluation.

## Methods

### Study design and participant recruitment

The data came from family members/partners of patients with different health conditions recruited through an online cross-sectional study conducted between April and November 2021. Participants were recruited through 58 UK-based patient support groups, research support platforms (Healthwise Wales-[HWW] [22], Autism Research Centre-Cambridge University database [ARC], Join Dementia Research [JDR]) and the Welsh social services departments. Family members/partners of patients completed the FROM-16 and EQ-5D-3L questionnaires. Ethics approval was given by the Cardiff University School of Medicine Research Ethics committee (SREC reference: 21/19), conforming to the principles embodied in the Declaration of Helsinki. The study was open to family members/partners (aged ≥ 18 years) of patients with any health condition and any age or gender living in the UK.

### Measures

#### FROM-16

The FROM-16 is a generic family QoL questionnaire which measures the impact of any disease, across all medical specialities, on the QoL of adult family members or partners of patients of any age [13]. The FROM-16 comprises 16 items, each with three response options: ‘Not at All’ (scoring 0), ‘A Little’ (scoring 1) and ‘A Lot’ (scoring 2). The lowest possible score of FROM-16 is 0, and the highest 32. The higher the score, the more negative the family member’s QoL.

#### EQ-5D-3L

The Euroqol EQ-5D is a generic HRQoL questionnaire which measures preferences associated with a particular health state. The EQ-5D consists of 5 dimensions (mobility, self-care, usual activities, pain, and anxiety), each with 3 levels (no problem, some problems, and extreme problems) coded 1 to 3. The EQ-5D-3L descriptive system presents 243 health states that are combined to calculate a single index, where the best health status is "11111", and the worst "33333". For this study, the index was calculated using the set of specific values (Tariffs) of the EQ-5D-3L UK version [23]. In this tariff, the utility values attached to different EQ-5D health states range from − 0.594 to 1, where 1 is defined as perfect health, 0 represents death, and negative values denote health states worse than death.

### Statistical analysis

#### Exploratory analysis

The frequencies and percentages of each response category of the items of both questionnaires were calculated along with the mean and standard deviation (SD) for the continuous variables. The distributions of the EQ-5D-3L index and FROM-16 dimensions were graphically observed through histograms, and normality was checked using Shapiro–Wilk’s test. Spearman correlations between the EQ-5D-3L index and the FROM-16 total score were calculated, defining “moderate” correlation as values between 0.30 and 0.49, “strong” between 0.50 and 0.69 and “very strong” for a value > 0.70 [24].

#### Mapping the FROM-16 to EQ-5D responses

We used the multinomial logistic regression (mlogit) to explore the association between individual FROM-16 responses (independent variable) and EQ-5D responses for each dimension (dependent variable). As the dependent variables are ordinal in nature, ordinal logistic regression would be the preferred method. However, the ordered logit model relies on an assumption of proportional odds or parallel regression, which means it generates a set of binary response models for the different ordered categories, in which the intercept varies, but the coefficients for the explanatory variables are the same. We first attempted ordinal logistic regression but found that the assumption of proportional odds was violated for all dimensions of EQ-5D-3L (the test for parallelism within SPSS gave significant results for all five EQ-5D dimensions, indicating violation of the proportional odds assumption). The alternative was therefore mlogit, which avoids the parallel regression assumption and provides unbiased parameter estimates. Using all data, a series of mlogit regressions were fitted for each of the five EQ-5D dimensions against the 16 individual items of FROM-16, as well as age and sex, using SPSS version 27. All 16 FROM-16 items were included for each domain model to capture all correlations induced by each FROM-16 item. Regressions were run with age and sex alone, FROM-16 items alone, as well as age and sex combined with FROM-16 items (Supplementary File 1, Table S1) to evaluate the contribution of age and sex, and collectively the FROM-16 items. Model comparisons were undertaken by comparing twice the absolute difference in the maximized log-likelihoods with the Chi-square distribution, with degrees of freedom equal to the difference in the number of model terms being evaluated (Supplementary File 1, Table S1).

#### Split-half cross-validation

This study employed the Split-half method used by Ali et al. [25] for mapping the Dermatology Life Quality Index (DLQI) to the EQ-5D, whereby the dataset was randomly split five times into separate estimation and validation sets using the SPSS version 27 random number generator. The estimation set was used to derive the mapping models, whilst the out-of-sample validation set was utilised for validating the fitted models. The multinomial logistic regression was conducted for each pair of datasets using FROM-16 items, age, and sex as independent variables. The model was tested on each validation dataset to produce three predicted probabilities per subject per EQ-5D domain (*Y* = 1, 2, or 3). Using these predicted probabilities, a Monte Carlo simulation was carried out for each subject resulting in predicted domain responses and consequently health utility estimates. A Monte Carlo method ensures that unbiased expected values are obtained and allows individuals to be identified within the EQ-5D descriptive system and predicted utility scores or tariffs to be calculated using the UK time trade off (TTO) values [23]. The five estimation and validation sets were then switched, and the process was repeated (split-half cross-validation), resulting in ten models. The average predicted health utility estimate for each validation set was then compared with the observed health utility estimate of the same set. Means square error (MSE) and mean absolute error (MAE) were compared and averaged across 10 validation models. The final model algorithm was based on the entire sample of data from 4228 family members/partners [26].

## Results

### Study sample demographic characteristics

A total of 4228 family members/partners of patients across 27 medical specialities, mostly from England and Wales, completed the EQ-5D and FROM-16 questionnaires (Table 1, Supplementary File 1, Table S2a). The mean age of family members was 57.7 (SD = 14.2) years, 65% were female. Patients’ mean age was 61.6 (SD = 20.2) years, 54% female. The family members were mostly the patient’s spouse/partner (60%), sons/daughters (22%) and parents (12%) (Table 1).

**Table 1:** Demographics and descriptive statistics

Variables	*n* (%) or M (SD)
Family members (n = 4228)	
Gender	
Male	1479 (35.0%)
Female	2749 (65.0%)
Age (years)	
Mean (SD)	57.69 (14.2)
Median	60
Range (IQR)	18–95 (20)
Relationship to patient	
Spouse/Partner	2532 (59.9%)
Son/daughters	921 (21.7%)
Parent	503 (11.9%)
Other^a^	272 (6.4%)
Patients (n = 4228)	
Gender	
Male	1928 (45.6%)
Female	2290 (54.2%)
Prefer not to say	4 (0.1%)
Other	6 (0.1%)
Age (years)	
Mean (SD)	61.62 (20.2)
Median	66
Range (IQR)	2–100 (26)
Place of residence in UK	
England	1779 (42.1%)
Northern Ireland	45 (1.1%)
Scotland	175 (4.1%)
Wales	2229 (52.7%)
FROM-16 total score (n = 4228)	
Mean (SD)	14.79 (8.1)
Range	0–32
EQ-5D-3L utility score (n = 4228)	
Mean (SD)	0.673 (0.3)
Range	− 0.594–1
EQ-VAS (n = 4209)	
Mean (SD)	68.44 (21.9)
Range	0–100
FROM-16 & EQ-5D correlation	
r_s_	0.450*

*r*_*s*_ Spearman correlation coefficient

^a^Brother/Sister, Father/Mother in law, Grandparent, Uncle/Aunt, Grandson/Granddaughter, Brother/Sister in law, Nephew/Niece, Cousin, friend

*Significant at 1%

### FROM-16 and EQ-5D scores

The mean FROM-16 total summary score and the EQ-5D-3L utility score were 14.8 (SD = 8.1) and 0.673 (SD = 0.3) (Table 1). Among FROM-16 items, ‘feeling worried’ was the most rated impact and ‘effect on travel’ was the least rated impact, while on the EQ-5D-3L domains, anxiety/depression was the most rated problem and ‘selfcare’ was the least rated problem (Supplementary File 1, Tables S2b, S2c). There was no evidence of significant multicollinearity between the sixteen FROM-16 items. For example, the correlation coefficient between worry, anger, sadness and frustration ranged from 0.424 to 0.593, less than the 0.7 threshold for multicollinearity (Supplementary File 1, Table S2d).

### Characterising the distribution and conceptual overlap

**Fig. 1:**
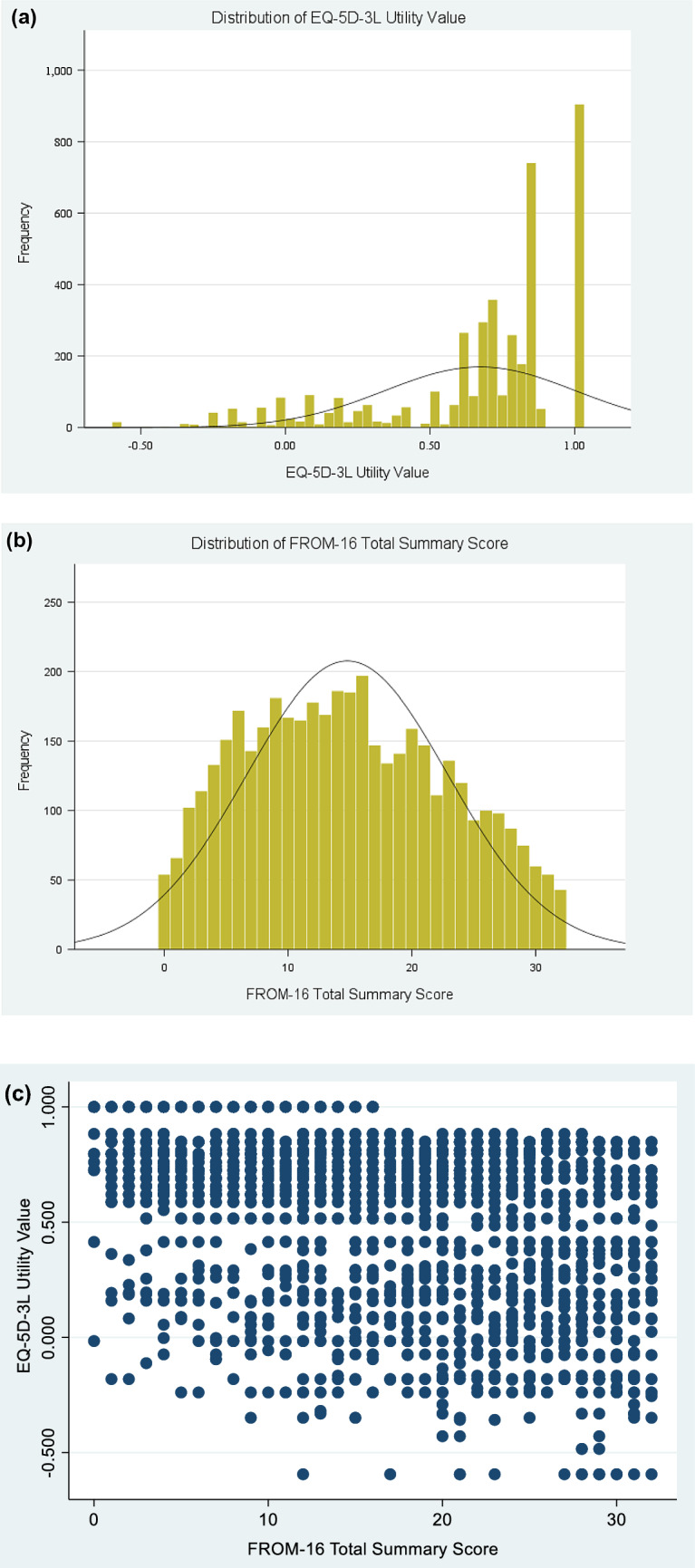
**a** Distributions of the EQ-5D-3L utility value; **b** Distributions of FROM-16 Total Summary Score; **c** Scatterplot showing the relationship between FROM-16 total summary score and EQ-5D utility value

Figure 1 shows the distribution plots of the EQ-5D-3L utility scores and FROM-16 total scores. FROM-16 appears to be normally distributed while EQ-5D-3L appears to be negatively skewed, indicating non-normality. Although the Shapiro–Wilk’s test was significant for FROM-16 and EQ-5D data sets indicating non-normality, for large sample sizes, histograms are more appropriate [27]. The correlation between the FROM-16 total summary score and the EQ-5D-3L utility scores was moderate with a Spearman’s rank correlation coefficient (r_s_) of 0.45. The EQ-5D anxiety/depression was strongly associated with FROM-16 domains (Emotional domain *r* = 0.52; Personal and social domains *r* = 0.50) while the EQ-5D mobility showed weakest association with FROM-16 Emotional domain (*r* = 0.132) (Supplementary File 1, Table S3). The relationship between FROM-16 summary scores and EQ-5D utility scores is shown in Fig. 1c (*r*_s_ = 0.45)”.

### Split-half cross-validation and model performance

Five times random split of the entire sample (*n* = 4228) into two parts resulted in five derivation and five validation sets of 2114 family members each. For each of the five EQ-5D domains, an mlogit model was derived and used to predict the probability of each EQ-5D response for each subject in each validation set using Monte Carlo simulation, and subsequently, the health utility was estimated. The predicted utilities for each validation set were compared to the observed utility. In each case, the predicted mean utility value was lower than the actual mean utility value indicating a slight overestimate of poor health (Table 2).

**Table 2:** Split-half cross validation using multinomial logistic regression: differences between actual and predicted utility value

Cross validation set	Actual utility			Predicted utility			Actual versus predicted		
	Mean (SD)	Min	Max	Mean (SD)	Min	Max	Diff in means	MSE	MAE
Set 1	0.667 (0.342)	− 0.594	1	0.662 (0.262)	− 0.239	1	0.006	0.135	0.267
Set 2	0.669 (0.331)	− 0.594	1	0.655 (0.277)	− 0.286	1	0.008	0.136	0.269
Set 3	0.673 (0.331)	− 0.594	1	0.646 (0.276)	− 0.237	1	0.027	0.140	0.275
Set 4	0.672 (0.326)	− 0.594	1	0.667 (0.277)	− 0.349	1	0.005	0.138	0.267
Set 5	0.680 (0.326)	− 0.594	1	0.654 (0.279)	− 0.429	1	0.027	0.132	0.266
Set 6	0.679 (0.320)	− 0.594	1	0.659 (0.274)	− 0.286	1	0.020	0.135	0.268
Set 7	0.677 (0.331)	− 0.594	1	0.648 (0.273)	− 0.222	1	0.029	0.140	0.274
Set 8	0.672 (0.330)	− 0.594	1	0.667 (0.278)	− 0.426	1	0.005	0.141	0.270
Set 9	0.674 (0.336)	− 0.594	1	0.657 (0.273)	− 0.322	1	0.017	0.138	0.269
Set 10	0.666 (0.336)	− 0.594	1	0.658 (0.273)	− 0.322	1	0.007	0.136	0.268
Average of 10 Sets	0.673 (0.331)	− 0.594	1	0.658 (0.274)	− 0.312	1	0.015	0.137	0.269

*SD* Standard deviation, *Diff in means* Difference in means, *MSE* Mean square error, *MAE* Mean absolute error

Across the ten validation sets, the difference between actual and predicted mean values ranged from 0.005 to 0.029, with an overall mean difference of 0.015. This 2.2% overestimate represents a clinically unimportant effect; the minimal clinically important difference of EQ-5D varies from 0.03 to 0.52 [28]. The mean square error (MSE) across all ten validation sets ranged from 0.132 to 0.141 (mean = 0.137), and the mean absolute error (MAE) across all ten validation sets ranged from 0.266 to 0.275 (mean = 0.269).

Table 3 reports error across subset range (EQ-5D < 0, 0 ≤ EQ-5D < 0.25, 0.25 ≤ EQ-5D < 0.5, 0.5 ≤ EQ-5D < 0.75,0.75 ≤ EQ-5D ≤ 1), to further understand variation between observed and predicted utilty values [26]. The smallest difference between observed and predicted mean (ME = − 0.007) was found for 0.25 ≤ EQ-5D subset while the largest difference (ME = − 0.079) in mean was for subset EQ-5D < 0. This is consistent with the finding that the degree of error is not evenly distributed across the scale of the dependent variable with overall the level of error being far greater at the lower (more severe health state) end [29].

**Table 3:** Comparison of observed and predicted utilities for EQ-5D subset range

	Mean EQ-5D-3L					
	Obs/Pre	< 0	0 to < 0.25	0.25– < 0.5	0.5– < 0.75	≤ 0.75
Set 1	Obs	− 0.159	0.122	0.341	0.662	0.9
	Pre	− 0.065	0.150	0.341	0.678	0.86
Set 2	Obs	− 0.153	0.126	0.333	0.662	0.897
	Pre	− 0.086	0.151	0.338	0.681	0.866
Set 3	Obs	− 0.173	0.131	0.335	0.660	0.897
	Pre	− 0.068	0.152	0.347	0.683	0.864
Set 4	Obs	− 0.162	0.128	0.331	0.658	0.896
	Pre	− 0.078	0.147	0.356	0.692	0.867
Set 5	Obs	− 0.153	0.128	0.340	0.658	0.899
	Pre	− 0.095	0.148	0.350	0.685	0.865
Set 6	Obs	− 0.153	0.132	0.339	0.661	0.898
	Pre	− 0.084	0.151	0.344	0.682	0.865
Set 7	Obs	− 0.158	0.128	0.346	0.661	0.901
	Pre	− 0.060	0.148	0.349	0.680	0.857
Set 8	Obs	− 0.139	0.123	0.344	0.663	0.901
	Pre	− 0.073	0.147	0.352	0.683	0.870
Set 9	Obs	− 0.151	0.126	0.351	0.665	0.902
	Pre	− 0.086	0.151	0.345	0.681	0.862
Set 10	Obs	− 0.159	0.126	0.340	0.665	0.899
	Pre	− 0.077	0.151	0.343	0.683	0.865
Mean across 10 sets	Obs	− 0.156	0.127	0.340	0.662	0.899
	Pre	− 0.077	0.149	0.347	0.683	0.864
Mean Error (ME) across 10 sets		− 0.079	− 0.022	− 0.007	− 0.021	0.035

To test the predictive performance of the equations, EQ-5D responses were assigned using a Monte Carlo approach in which random numbers were compared with the probability values estimated by the mlogit models. Using all FROM-16 questions, age and gender as predictors, the overall proportion of predicted responses allocated to the correct level varied across ten models with most (79%) having 90–100% accuracy. Accuracy was less than 70% (Supplementary File 1, Table S4) in only 4% of responses. In general, predicted levels that were ‘off-diagonal’ were equally likely to be lower or higher than the actual level (Supplementary File 1, Fig. S1).

**Fig. 2:**
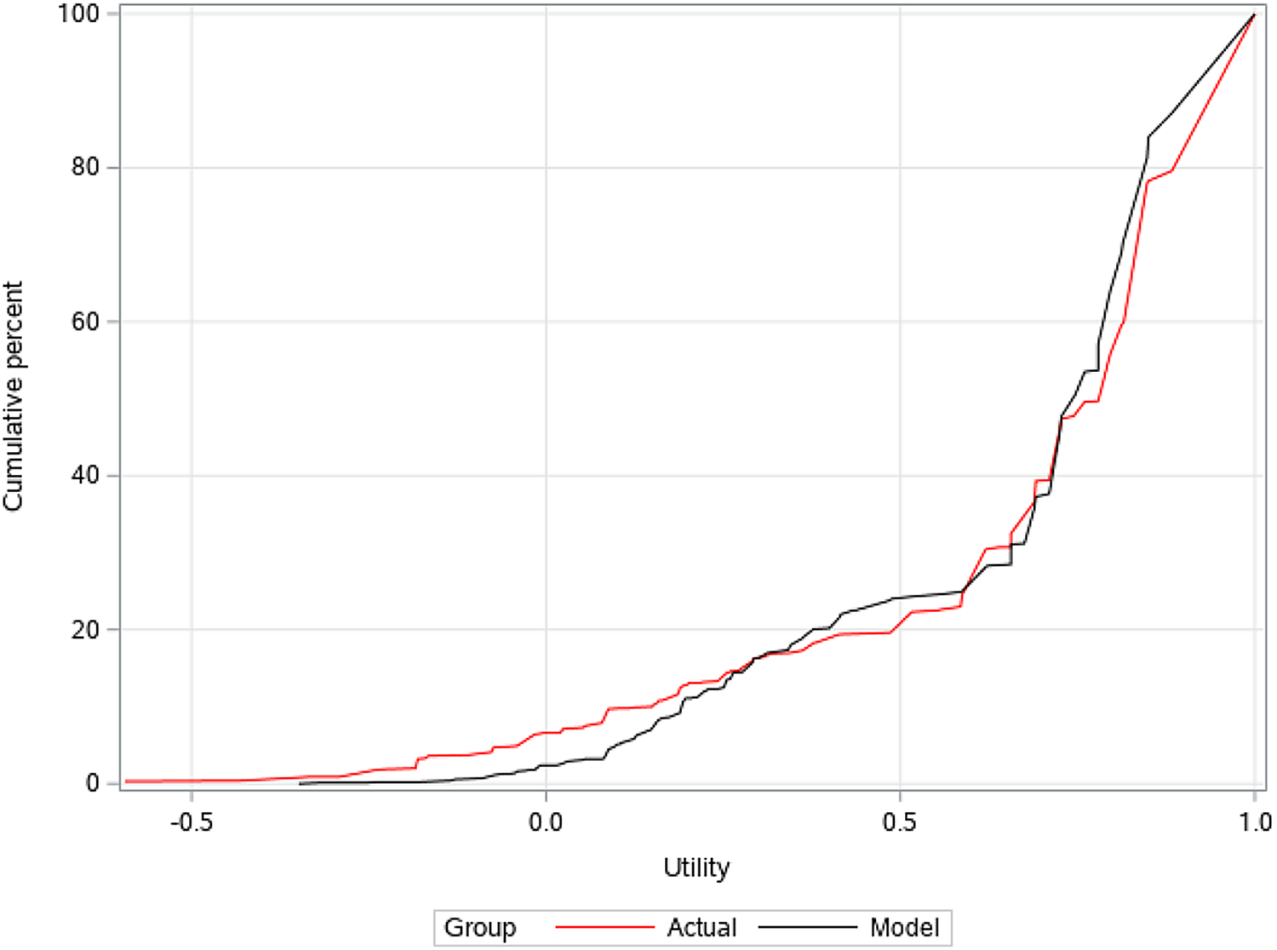
The cumulative percentage of observed EQ-5D-3L utility values vs. simulated values for a typical model (model 4/10)

**Fig. 3:**
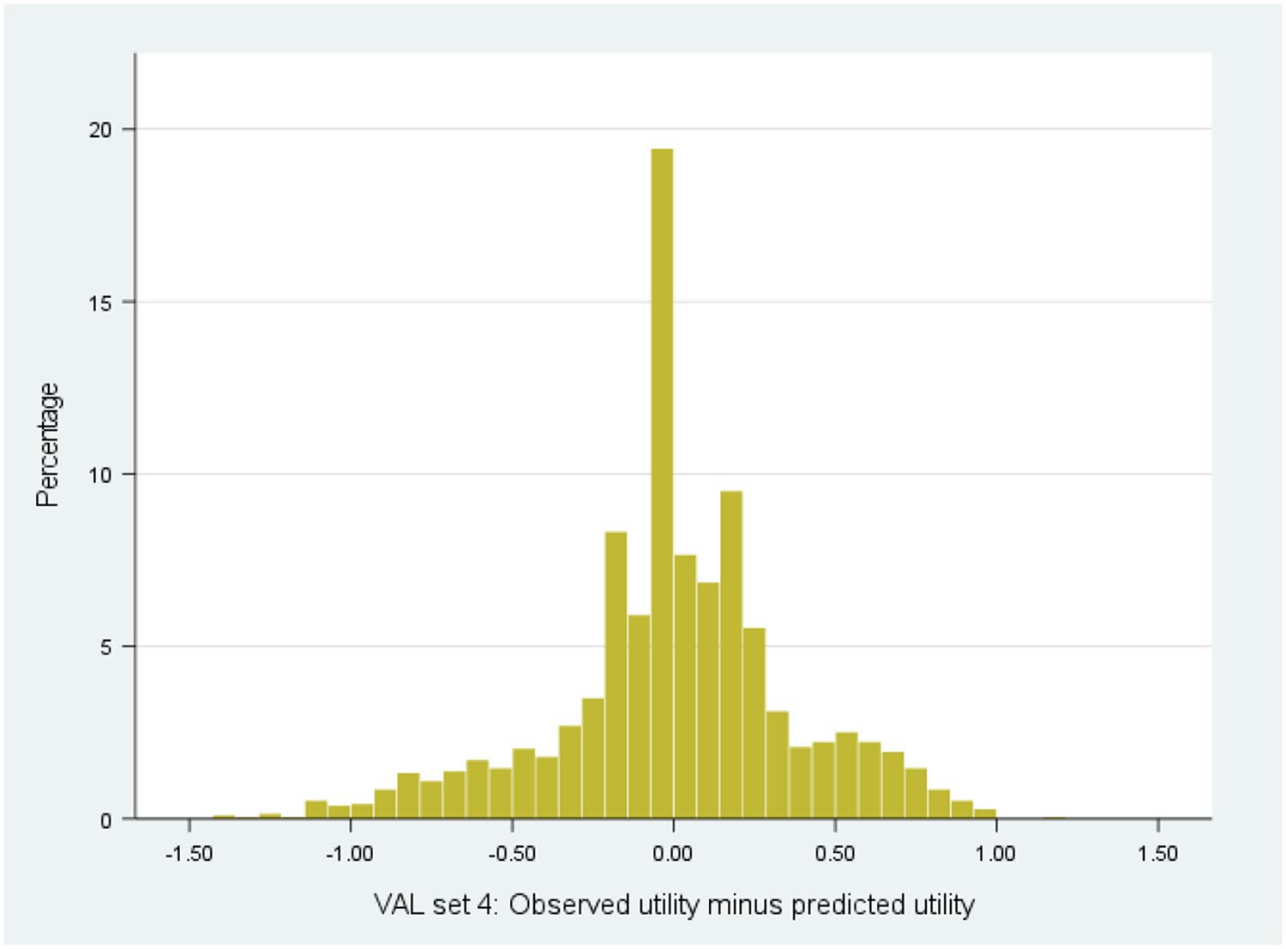
Histogram displaying the difference between predicted and observed health utility estimates for a typical cross validation set 4

To further compare results, we examined cumulative distribution of observed and predicted utility data across ten validation models (Fig. 2). For models 2, 4, 6 and 8 the predicted distribution was closer to observed data than the other models (Supplementary File 1, Fig. S2). The model was shown to be highly predictive and repeated data splits demonstrated its stability (Table 2). The predictive ability of the model at an individual subject level was examined using histograms to display the difference between the predicted utility score and the actual utility score for each simulation for individual subjects (Fig. 3). The results from all ten splits are displayed in Supplementary File 1, Fig. S3.

All the plots depict a centrality around ‘0’, indicating the strong predictive collective capability of the mlogit models. On average across ten validation models, 54% of the individual utility values were predicted to lie within 0.05 of the actual values, 59% within 0.1, 73% within 0.2 and 83% within 0.3 of the actual values (Supplementary File 1, Table S5).

We also explored the Expected utility method [30]) which uses predicted probabilities of response levels to directly calculate utility as opposed to a simulated distribution with the Monte Carlo simulation method (Supplementary File 1, Table S6). We found that SDs, MSE and MAE values were even less for the Expected utility method than those found for the Monte Carlo simulation method (Table 2) [30]. However, 7.2% of the sample had a utility value less than “0” (worse than death on EQ-5D scale) and 21.4% of the sample had a utility value of “1” (perfect health on the EQ-5D scale), but these values could not be predicted using the Expected utility method.

The final algorithm was derived from the entire sample of 4228 family members using Monte Carlo simulation. Details of the final-fitted mlogit models using data from the 4228 family members are given in Table 4. This Table shows the regression coefficients, which can be used to estimate the probability that a respondent will select a particular level of response to questions in the EQ-5D, using individual question responses from the FROM-16 as predictors. The coefficients for individual variables in this type of model are not straightforward to interpret but the results indicate that the FROM-16 question responses most likely to be related to EQ-5D question responses are generally highly significant; for example, responses to the FROM-16 ‘effect on family activities’ question are significantly related to the responses to the EQ-5D ‘usual activities’ question but not to responses to the EQ-5D ‘mobility’, ‘self-care’, ‘pain/discomfort’ or ‘anxiety’ questions; similarly, the FROM-16 responses to the ‘effect on family relationship’ question are significantly related to the responses to the EQ-5D ‘anxiety’ question. This is understandable as the impact on a relative of caring, with its effect on family relationships, could contribute to a relative’s anxiety and depression. An example showing how to use the FROM-16 mapping algorithm is provided in Supplementary File 2﻿﻿.

**Table 4:** Final model coefficients (standard errors) for each EQ-5D domain against 16 items of FROM-16, age and gender using multinomial logistic regression

	Mobility		Self-care		Usual activities		Pain/discomfort		Anxiety/depression	
	Some problems	Extreme problems	Some problems	Extreme problems	Some problems	Extreme problems	Some problems	Extreme problems	Some problems	Extreme problems
Intercept	− 3.468 (0.22)*	− 7.488 (0.962)*	− 3.25 (0.28)*	− 6.497 (0.732)*	− 2.746 (0.206)*	− 5.344 (0.422)*	− 2.18 (0.191)*	− 4.437 (0.357)*	− 1.434 (0.205)*	− 4.012 (0.377)*
Age	0.031 (0.003)*	0.008 (0.011)	0.004 (0.004)	0.000 (0.008)	0.019 (0.003)*	0.009 (0.005)	0.026 (0.003)*	0.017 (0.005)*	− 0.013 (0.003)*	− 0.032 (0.005)*
Gender^a^	− 0.05 (0.079)	− 0.059 (0.332)	− 0.297 (0.105)*	0.153 (0.262)	− 0.089 (0.079)	− 0.205 (0.148)	− 0.008 (0.075)	− 0.247 (0.131)	0.269 (0.083)*	− 0.037 (0.143)
FROM_Worried	0.276 (0.078)*	− 0.14 (0.347)	0.387 (0.11)*	− 0.227 (0.26)	0.17 (0.076)*	0.255 (0.158)	0.17 (0.072)*	0.383 (0.138)*	0.522 (0.077)*	0.715 (0.151)*
FROM_Angry	0.094 (0.063)	0.288 (0.236)	0.009 (0.082)	0.221 (0.182)	0.042 (0.062)	0.137 (0.11)	0.081 (0.061)	0.129 (0.101)	0.269 (0.069)*	0.395 (0.107)*
FROM_Sad	− 0.303 (0.067)*	0.346 (0.334)	− 0.136 (0.092)	0.624 (0.254)*	− 0.211 (0.066)*	0.264 (0.138)*	− 0.328 (0.063)	− 0.352 (0.116)*	0.117 (0.068)	0.353 (0.129)*
FROM_Frustrated	− 0.071 (0.067)	− 0.078 (0.307)	− 0.024 (0.092)	− 0.525 (0.22)*	− 0.042 (0.065)*	− 0.266 (0.13)*	0.025 (0.062)	0.01 (0.115)	− 0.021 (0.067)	− 0.085 (0.124)
FROM_Talking thoughts	0.199 (0.055)*	0.458 (0.23)*	0.132 (0.073)	0.172 (0.168)	0.203 (0.053)	0.192 (0.099)*	0.134 (0.052)*	0.323 (0.09)*	0.403 (0.058)*	0.777 (0.097)*
FROM_Difficulty caring	0.002 (0.069)	0.341 (0.304)	0.064 (0.091)	0.68 (0.244)*	0.09 (0.066)	0.301 (0.13)*	− 0.121 (0.064)*	0.085 (0.114)	0.029 (0.071)	0.223 (0.12)
FROM_Time for self	− 0.066 (0.07)	0.204 (0.277)	− 0.159 (0.093)	0.124 (0.219)	− 0.038 (0.068)	0.228 (0.127)	0.022 (0.067)	− 0.267 (0.113)*	0.244 (0.075)*	0.356 (0.12)*
FROM_Travel	0.152 (0.061)*	0.018 (0.207)	0.187 (0.078)*	0.183 (0.164)	0.161 (0.06)*	0.313 (0.1)*	0.093 (0.06)	0.305 (0.096)*	0.102 (0.072)	0.154 (0.105)
FROM_Eating habits	0.221 (0.061)*	0.15 (0.218)	0.292 (0.078)*	0.141 (0.168)	0.107 (0.06)	0.202 (0.103)*	0.094 (0.06)	0.364 (0.095)*	0.198 (0.071)*	0.613 (0.104)*
FROM_Family activities	0.083 (0.071)	0.211 (0.328)	0.031 (0.096)	0.149 (0.256)	0.19 (0.068)*	0.194 (0.141)	0.092 (0.066)	0.035 (0.12)	0.1 (0.072)	0.059 (0.128)
FROM_Holiday	− 0.004 (0.063)	0.249 (0.287)	− 0.08 (0.086)	0.275 (0.228)	0.027 (0.061)	0.083 (0.124)	0.002 (0.059)	− 0.027 (0.107)	0.047 (0.065)	− 0.214 (0.112)*
FROM_Sex life	0.095 (0.047)*	− 0.06 (0.177)	0.119 (0.063)*	0.000 (0.14)	0.109 (0.046)*	0.202 (0.085)*	0.116 (0.045)*	0.131 (0.076)	0.069 (0.051)	0.073 (0.082)
FROM_Work or study	− 0.241 (0.065)*	− 0.152 (0.209)	− 0.262 (0.082)*	0.227 (0.168)	0.037 (0.064)	0.032 (0.103)	− 0.133 (0.064)*	− 0.329 (0.099)*	0.031 (0.077)	− 0.028 (0.107)
FROM_Family relationships	− 0.072 (0.065)	0.455 (0.25)	0.068 (0.085)	0.081 (0.185)	− 0.005 (0.064)	0.044 (0.111)	0.076 (0.062)	− 0.063 (0.103)	0.21 (0.071)*	0.277 (0.109)*
FROM_Family expenses	0.333 (0.058)*	0.479 (0.231)*	0.346 (0.077)*	0.563 (0.182)*	0.3 (0.056)*	0.395 (0.103)*	0.239 (0.055)*	0.527 (0.095)*	− 0.083 (0.063)	0.082 (0.1)
FROM_Sleep	0.127 (0.065)	− 0.16 (0.28)	0.223 (0.089)*	− 0.258 (0.213)	0.195 (0.063)*	0.133 (0.126)	0.151 (0.061)*	0.598 (0.113)*	0.404 (0.067)*	0.704 (0.12)*

FROM-16 items represented as FROM_item name; No problem is the comparison group

^a^Gender was coded male = 0, female = 1

*Significant at 5%

## Discussion

This mapping of a generic family QoL measure to EQ-5D, facilitates conversion of family member and/or informal carer’s QoL scores into utility values for health economic evaluation. Over six million family members in the UK care for relatives with health conditions [31], with major impact on their QoL [32–35]. However, a major gap in the inclusion of family members in utility analysis may be caused by lack of family member/informal carer data [4]. As value-sets exist for Carerqol-7D and carers’ utility can be assessed directly, perhaps CarerQol-7D use could be prioritised. However, CarerQol-7D, a care-related QoL measure, encompasses dimensions such as support and fulfilment and therefore its scores cannot be summated with patient utilities derived from a health-related utility measure such as EQ-5D-3L [8]. As there is no carer equivalent to EQ-5D, NICE has used EQ-5D to measure carer utility, however, EQ-5D may be inappropriate for family member/informal carers [7]. For example, the EQ-5D question on ‘mobility as a moderate effect’ may mean to family members an inability to go out to meet people, while ‘mobility as an extreme effect’ may confuse family caregivers as to why they should be ‘confined to bed’. EQ-5D asks general questions and not specific questions about the QoL impact of caring, such as on sleep, relationships and expenses. However, EQ-5D can still be used to assess family member/informal carer utility with some validity [11, 12]. FROM-16, based on the perspective of family members/partners of patients from 26 medical specialities [13] could be used for assessing family member/informal carer utility when EQ-5D data is unavailable. Perhaps measuring family member/informal carer impact might “double count” QoL impact, but effect on family members is a real additional impact [36].

The study used the method employed [25] for mapping DLQI scores to EQ-5D utility values and followed guidance concerning mapping to obtain EQ-5D utility values for use in NICE health technology assessments [26]. The study used the response mapping method to map FROM-16 responses to EQ-5D using multinomial logistic regression to predict probabilities and the Monte Carlo simulation method to generate predicted EQ-5D responses, the method first used to map SF-12 responses and EQ-5D utility values [15]. In our modelling, we used FROM-16 item scores as continuous independent variables. To have included FROM-16 items with categories may have resulted in only marginal improvements, given the complexity of running that model. Furthermore, it is not unusual to use item scores rather than categories as independent variables [25]. The mean observed utility across the ten validation sets was 0.67 (SD = 0.33), and the mean predicted utility was 0.66 (SD = 0.27), both considerably lower than the UK general population utility value of 0.83 (SD = 0.32) [37]. Since the sample was taken from family members of patients across > 200 different health conditions, this predicted utility already indicates the considerable QoL impact on the patients’ family members/partners. As data were collected during the COVID pandemic, difficulties experienced by family members caring for their relative might have contributed to lower utility values. However, our aim was to create equivalence to EQ-5D utility values rather than estimating burden. Most (65%) family members/partners were female, representative of the UK gender distribution of carers (68% females) [31, 38].

In this study, the mean difference between observed and predicted utility across ten validation sets was 0.015, indicating a slight but clinically unimportant overestimate of poor health. The MSE across ten validation sets ranged from 0.132 to 0.141 (average = 0.137), and the MAE ranged from 0.266 to 0.275 (average = 0.269). Although the mean errors MSE and MAE were slightly higher than in the DLQI mapping study [25] (MSE = 0.073–0.082, mean across 10 sets = 0.077; MAE = 0.187–0.201; mean across 10 sets = 0.193), we are modelling a family-specific measure to EQ-5D, hence such variation is expected. Compared to direct methods, the response mapping method is penalised for any incorrect prediction leading to increased MSE [19, 39].

The model reliably predicts EQ-5D scores, especially at group level, demonstrated through a split-half cross-validation process resulting in very close health utility estimate predictions. On average, 54% of the individual utility differences were predicted to lie within 0.05 of the actual values: this is comparable to Gray et al.’s findings [19]. 59.12% were predicted to lie within 0.1, 73% within 0.2 and 83% were within 0.3 of actual values. These are still important differences on a scale of 0–1, but the model’s group-level performance demonstrates better predictive ability. Overall predictions were strongly correlated to the observed scores at a group level, the model’s predicting power at individual level requires further evaluation. Other mapping studies with similar results [15, 25] have recommended interpreting results at a group level.

For successful mapping, there should be conceptual overlap between the source and target instruments [40]. There were significant correlations between the FROM-16 domains and EQ-5D domains, with emotional domain strongly correlated to anxiety/depression followed by activity, self-care, pain, and mobility. The personal and social domain of FROM-16 was also strongly correlated to anxiety/depression, followed by activity, pain/discomfort, self-care, and mobility.

If an external dataset is not available to assess performance of a predicted model, random splitting of the sample into an estimation sample and validation sample is recommended. This does not result in true randomisation and may result in statistical bias if data is only split once [25]. The Split half-cross validation method [20] used in this study overcomes this disadvantage, improves the accuracy of the model and demonstrates that the predicted utility values accuracy is not due to chance [25]. This method may reduce the sample size of the estimation sample leading to reduced precision. Although our sample was large enough not to be affected by splitting of data, the final model algorithm was based on the entire data sample from 4228 family members/partners [26]. As our sample came from a UK population of family members/partners across 27 medical specialities and a wide range of condition severities, we believe our model is generalisable to the UK population.

We used the response mapping approach which follows the EQ-5D logic by predicting health states and attaching utility tariff values to these. This allows predicted response values to be used in different countries using a country-specific tariff, important as values derived from a UK value set tend to be lower than for other countries [39]. Cultural attitudes might influence HRQoL and utility responses, but a model created on an Italian population worked equally well on a Norway population [25].

When mapping between measures, lack of accuracy in data and lack of test–retest reliability may result in bias. Use in analyses of incremental treatment effects increases the risk of making wrong recommendations about the cost-effectiveness of treatments. This can be minimised by measure developers applying appropriate reliability tests. FROM-16 is responsive to changes in family members’/partners’ health-related QoL over time [41], indicating that it can reliably measure changes in family members’ QoL. Although mapping of FROM-16 to EQ-5D has shown that FROM-16 can reliably predict EQ-5D scores, we do not have evidence that mapping would produce better estimates. Using utility values generated through mapping is most appropriate when EQ-5D data is not available, as applied by NICE [42].

This study has several strengths. It is the first to explore the relationship between EQ-5D and FROM-16. Although EQ-5D has been mapped to patient generic measures [19], and disease specific measures [15, 21, 39, 43–45], this is the first to map EQ-5D to a family specific measure. The data in this study are representative of family members caring for their relative across all areas of medicine.

To justify including carer HRQoL in economic evaluation, the health condition should be associated with a substantial impact on a caregiver’s health and well-being [46]. Caregiver QoL should be assessed using the EQ-5D to be consistent with patient QoL data and to enable comparisons between appraisals [10, 46]. This study demonstrates that FROM-16 could be an excellent measure to capture this data and associated EQ-5D utilities across all health conditions.

There are study limitations. Firstly, no external sample dataset was available for external validation, as unlike patient reported outcomes [47], family outcomes are not regularly measured. Therefore, even though this study demonstrated how well the model performs outside of the sample, external validation with a different dataset of family members is recommended. If resources are available, and family members willing, FROM-16 and EQ-5D data should be collected directly, though mapping may sometimes be required. The robustness of the mapping model proposed should be further validated in long-term studies.

Inclusion of wider socio-demographic variables might improve the models’ predictive performance, but give only marginal improvements, not outweighing the complexity of running the model [19]. Our study sample included a high proportion of family members of patients with neurological conditions: this may have resulted in bias.

Accessible versions of our algorithms in a Microsoft Excel spreadsheet with pre-programmed formulae to enable EQ-5D domain probability calculations and health utility estimates from responses to FROM-16 are available on request from the authors.

## Conclusions

Although inclusion of evaluation of burden of family members/informal carers is encouraged by health economists and decision scientists, this seldom happens, primarily due to lack of family member/informal carer data. Our study fills this important research gap by mapping EQ-5D utility values to FROM-16, a generic instrument which can be used across all disease areas to measure the impact of patients’ disease on their family members’ QoL. The algorithm developed can be used by decision scientists and researchers to calculate EQ-5D-3L utility values from the FROM-16 scores when EQ-5D data is not available, thus allowing the inclusion of the value of the impact on the QoL of family members/informal carers in health technology appraisal.

### Supplementary Information

Below is the link to the electronic supplementary material.

Supplementary file1 (PDF 654 KB)

Supplementary file2 (PDF 395 KB)

## Data Availability

The data are available from the authors on reasonable request from authors according to Cardiff University regulations.
