# Supplementary material for: Mapping of Family Reported Outcome Measure (FROM-16) scores to EQ-5D: algorithm to calculate utility values
Source: Qual Life Res. 2024 Feb 25;33(4):1107–19. doi: 10.1007/s11136-023-03590-z (PMC10973087; doi:10.1007/s11136-023-03590-z)
Supplement: Supplementary file 1 — Supplementary file1 (PDF 654 KB) [file 11136_2023_3590_MOESM1_ESM.pdf]

Supplementary File 1

Mapping of Family Reported Outcome Measure (FROM-16) scores to EQ-5D: Algorithm to calculate Utility Values

Shah RM, Salek MS, Finlay AY, Kay R, Nixon SJ, Otwombe K, Ali MF, Ingram J

Corresponding author:

ShahR45@cardiff.ac.uk

INDEX: Tables/figures included in the online resource

Table S1 FROM-16 items compared to EQ-5D domains
Table S2a Descriptive statistics: Medical specialties included in the study
Table S2b EQ-5D-3L dimension scores (n=4228)
Table S2c FROM-16 Individual item scores
Table S2d Spearman's rho Correlations showing relationship between different items and testing multicollinearity#
Table S3 Correlation between FROM -16 and EQ-5D-3L
Table S4 Predicted EQ-5D responses across 10 cross validation Sets
Table S5 Mean utility value difference across all ten cross validation sets
Table S6 Actual utility values to predicted utility values across ten models (n=2114) using the Expected utility method

Figure S1 Comparison of predicted and observed EQ-5D-3L responses across ten validation sets
Figure S2 (a)-(j) The cumulative percentage of observed EQ-5D-3L data vs. simulated data across ten validation sets.
Figure S3 (a)-(j) Histograms demonstrating the mean difference between predicted and actual utility scores for each Monte Carlo simulation

T**able S1** FROM-16 items compared to EQ-5D domains*

EQ-5D Domain	Covariates: age/sex			Covariates: FROM-16 items			Covariates: age/sex/FROM-16 items		
	-2 log likelihood	Chi-square comparing to full model	Degrees of freedom (df)	-2 log likelihood	Chi-square comparing to full model	Degrees of freedom (df)	-2 log likelihood	Chi-square comparing to full model	Degrees of freedom (df)
Mobility	781.6	151.9	4	4946.9	341.9	32	5173.7	466.1	36
Selfcare	744.8	12.6	4	3582.8	342.2	32	3772.6	352.6	36
Usual activities	1049.8	50.7	4	6258.6	712.0	32	6589.8	764.1	36
Pain and discomfort	1068.3	114.8	4	6885.2	448.4	32	7279.9	551.8	36
Anxiety and depression	1078.1	177.3	4	5975.6	1642.9	32	6298.1	1703.2	36

**The significance of the FROM-16 items and age and sex compared to the model containing age, sex, and the FROM-16 items for each EQ-5D domain*

Table S2a *Descriptive statistics: Medical specialties included in the study*

**Patients (n=4228)**		**Number (%)**
Medical specialities	Audiology	19 (0.4)
	Cardiology	239 (5.7)
	Chronic pain	7 (0.2)
	Critical care	1 (0.02)
	Dermatology	127 (3.0)
	Endocrinology	266 (6.3)
	Gastroenterology	151(3.6)
	Genetic/ Rare disease	44 (1.0)
	Gynaecology	37 (0.9)
	Haematology	179 (4.2)
	Hepatology	10 (0.2)
	Immunology	12 (0.3)
	Infectious diseases	10 (0.2)
	Movement disorder	10 (0.2)
	Nephrology	55 (1.3)
	Neurology	1522 (36.0)
	Oncology	241 (5.7)
	Ophthalmology	89 (2.1)
	Orthopaedics	24 (0.6)
	Otolaryngology	6 (0.1)
	Rehabilitation medicine	30 (0.7)
	Paediatrics	134 (3.2)
	Psychiatry	311 (7.4)
	Respiratory medicine	261 (6.2)
	Rheumatology	302 (7.1)
	Urology	21 (0.5)
	Wound healing	1 (0.02)
	Multiple health conditions	91(2.2)
	Not stated	28 (0.7)

Table S2b *EQ-5D-3L dimension scores (n=4228)*

EQ-5D dimensions	No problems N (%)	Some problems N (%)	Extreme problems N (%)
Mobility	2939 (69.5)	1231 (29.1)	58 (1.4)
Selfcare	3588 (84.9)	540 (12.8)	100 (2.4)
Usual activity	2468 (58.4)	1447 (34.2)	313 (7.4)
Pain and discomfort	2063 (48.8)	1780 (42.1)	385 (9.1)
Anxiety and depression	1618 (38.3)	2157 (51)	453 (10.7)

Table S2c *FROM-16 Individual item scores*

FROM-16 item	Not at All N (%)	A little N (%)	A lot N (%)
Worried	239 (5.7)	1805 (42.7)	2184 (51.7)
Angry	1878 (44.4)	1638 (38.7)	712 (16.8)
Sad	645 (15.3)	1769 (41.8)	1814 (42.9)
Frustrated	791 (18.7)	1710 (40.4)	1727 (40.8)
Talking about Thoughts	1599 (37.8)	1421 (33.6)	1208 (28.6)
Difficulty caring	1179 (27.9)	1748 (41.3)	1301 (30.8)
Time for self	1660 (39.3)	1543 (36.5)	1025 (24.2)
Travel	2553 (60.4)	991 (23.4)	684 (16.2)
Eating habits	2425 (57.4)	1242 (29.4)	561 (13.3)
Family activities	902 (21.3)	1821 (43.1)	1505 (35.6)
Holiday	1341 (31.7)	1294 (30.6)	1593 (37.7)
Sex life	1901 (45)	944 (22.3)	1383 (32.7)
Work/study	2496 (59)	1127 (26.7)	605 (14.3)
Family relationships	1929 (45.6)	1530 (36.2)	769 (18.2)
Family expenses	1769 (41.8)	1460 (34.5)	999 (23.6)
Sleep	1233 (29.2)	1644 (38.9)	1351 (32)

Table S2d *Spearman's rho Correlations showing relationship between different items and testing multicollinearity*[#]

**FROM-16 Items**	Worried	Angry	Sad	Frustrated	Talking about thoughts	Difficulty caring	Time for self	Travel	Eating habits	Family activities	Holiday	Sexlife	Work /study	Family relationships	Family expenses	Sleep
Worried	1	.	.													
Angry	.424**	1														
Sad	.534**	.482**	1													
Frustrated	.453**	.573**	.497**	1												
Talking about thoughts	.411**	.453**	.383**	.444**	1											
Difficulty caring	.465**	.454**	.471**	.501**	.444**	1										
Time for self	.398**	.373**	.394**	.406**	.440**	.619**	1									
Travel	.285**	.284**	.263**	.314**	.328**	.443**	.557**	1								
Eating habits	.283**	.305**	.260**	.313**	.342**	.388**	.455**	.437**	1							
Family activities	.364**	.355**	.389**	.421**	.385**	.511**	.567**	.501**	.455**	1						
Holiday	.352**	.332**	.342**	.383**	.370**	.497**	.584**	.532**	.418**	.633**	1					
Sex life	.209**	.228**	.186**	.239**	.290**	.237**	.332**	.320**	.322**	.356**	.415**	1				
Work/study	.335**	.339**	.341**	.339**	.361**	.446**	.505**	.418**	.414**	.461**	.411**	.290**	1			
Family relationships	.392**	.397**	.420**	.405**	.426**	.548**	.528**	.406**	.412**	.512**	.435**	.222**	.511**	1		
Family expenses	.341**	.334**	.334**	.358**	.348**	.462**	.501**	.437**	.403**	.479**	.480**	.273**	.426**	.450**	1	
Sleep	.462**	.418**	.436**	.424**	.459**	.510**	.566**	.442**	.481**	.507**	.513**	.384**	.451**	.486**	.492**	1

** Correlation is significant at the 0.01 level (2-tailed). [#]Multicollinearity is present when Correlation coefficient >7

Table S3 *Correlation*[†] *between FROM -16 and EQ-5D-3L*

**FROM-16**	**Correlation to EQ-5D**	**r**
Emotional domain (item 1-6)	Anxiety/depression> Activity>Self care>Pain>mobility	0.523*>0.279*>0.176*>0.165*>0.132*
Personal and social (item 7-16)	Anxiety/depression>Activity> Pain>Self care> mobility	0.495*>0.367*>0.239*>0.220*>0.190*
FROM-16 total score	EQ-5D utility values	0.450*

† Spearman correlation; * p-value 0.05

Table S4 *Predicted EQ-5D responses across 10 cross validation Sets*

% of accuracy	Set 1	Set 2	Set 3	Set 4	Set 5	Set 6	Set 7	Set 8	Set 9	Set 10	Average
90-100%	80	86.66	86.66	86.66	86.66	73.33	73.33	73.33	73.33	73.33	79.33
70-100%	93.33	100	100	93.33	100	100	93.33	100	93.33	93.33	96.67
<70%	0.06	0.06	0	0.06	0	0	0.06	0	0.06	0.06	0.04

Table S5 *Mean utility value difference across all ten cross validation sets*

	Set 1	Set 2	Set 3	Set 4	Set 5	Set 6	Set 7	Set 8	Set 9	Set 10	Average
**Mean Utility Value Difference**	0.006	0.008	0.027	0.005	0.027	0.020	0.029	0.005	0.017	0.007	**0.015**
**% Utility Values within 0.05 of Actual UV**	53.6	54.6	51.8	58.6	53.3	53.8	51.6	56.6	54.3	54.5	**54.27**
**% Utility Values within 0.1 of Actual UV**	58.8	59.5	56.3	62.9	57.9	58.6	56.5	61.9	59	59.8	**59.12**
**% Utility Values within 0.2 of Actual UV**	73.4	73.9	70.2	74.6	73.3	72.8	71.3	75.4	71.8	73.6	**73.03**
**% Utility Values within 0.3 of Actual UV**	84.1	83	80.6	83.2	81.8	82.3	81.6	83.8	81.7	82.9	**82.50**

Table S6 Actual utility values to predicted utility values across ten models (n=2114) using the Expected utility method

Expected Utility Method*									
**Cross-validation Set**	**Actual Utility**			**Predicted Utility**			**Actual versus Predicted**		
	**Mean (SD)**	**Min**	**Max**	**Mean (SD)**	**Min**	**Max**	**Diff in means**	**MSE**	**MAE**
Set 1	0.667(0.342)	-0.594	1	0.653(0.159)	0.187	0.905	0.014	0.090	0.229
Set 2	0.669(0.331)	-0.594	1	0.655(0.166)	0.040	0.912	0.014	0.088	0.226
Set 3	0.673(0.331)	-0.594	1	0.637(0.161)	0.197	0.906	0.037	0.087	0.229
Set 4	0.672(0.326)	-0.594	1	0.657(0.164)	0.161	0.921	0.015	0.085	0.222
Set 5	0.680(0.326)	-0.594	1	0.645(0.162)	0.142	0.896	0.035	0.085	0.226
Set 6	0.679(0.320)	-0.594	1	0.648(0.163)	0.158	0.914	0.030	0.083	0.223
Set 7	0.677(0.331)	-0.594	1	0.643(0.160)	0.208	0.910	0.033	0.089	0.230
Set 8	0.672(0.330)	-0.594	1	0.660(0.166)	0.140	0.909	0.012	0.087	0.224
Set 9	0.674(0.336)	-0.594	1	0.654(0.152)	0.185	0.897	0.020	0.087	0.227
Set 10	0.666(0.336)	-0.594	1	0.656 (0.162)	0.204	0.921	0.010	0.090	0.227
**Average of 10 Sets**	0.673(0.331)	-0.594	1	0.651(0.162)	0.162	0.909	0.022	0.087	0.226

**Level 1 (No effect) was a reference category; SD, Standard Deviation; Min, Minimum; Max, Maximum; Diff in means , Difference in means; MSE, Mean Square Error; MAE, Mean Absolute Error.*

Fig S1 *Comparison of predicted and observed EQ-5D-3L responses across ten validation sets*

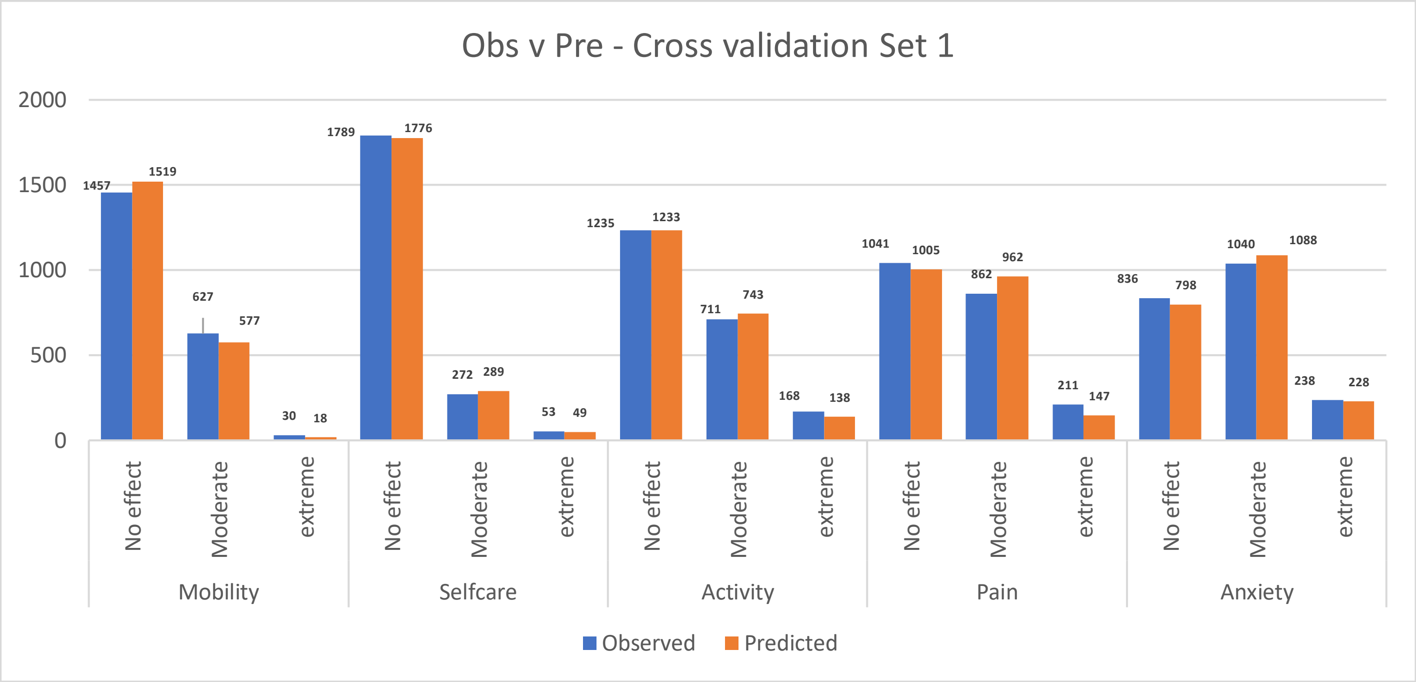

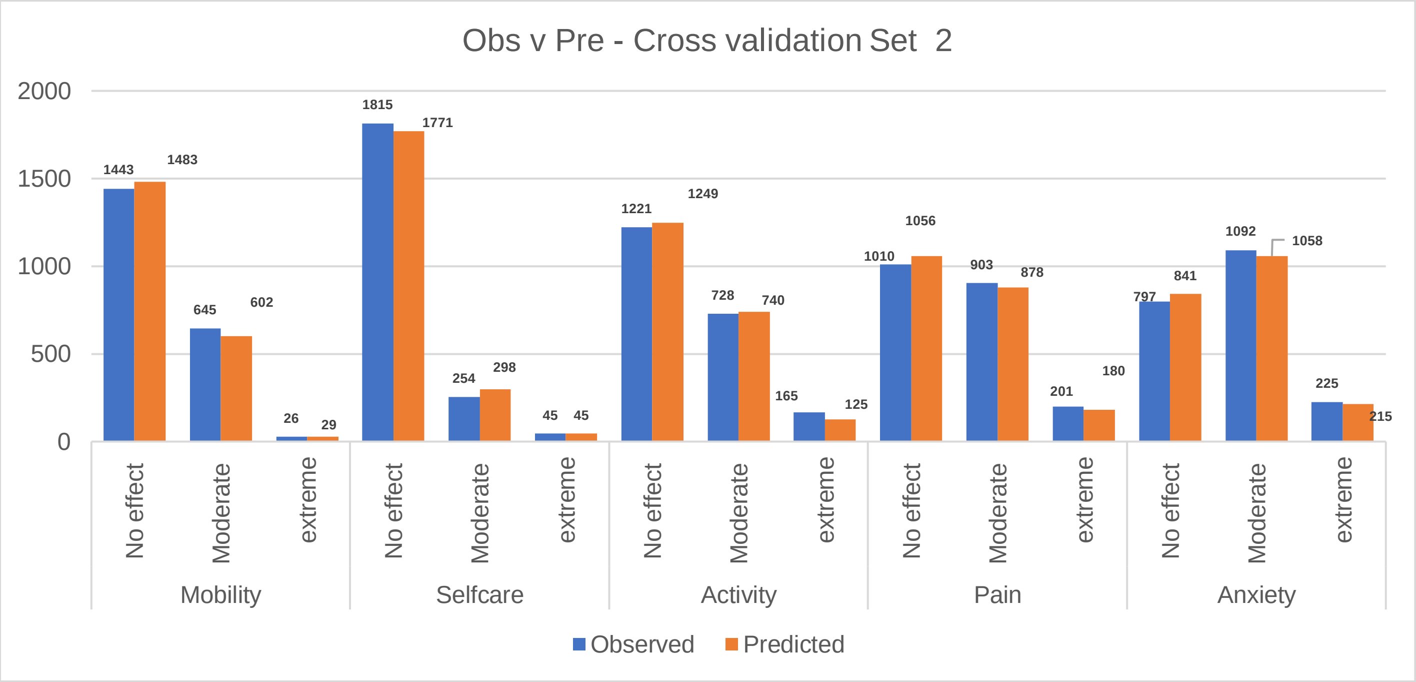

Fig S1 (Contd)

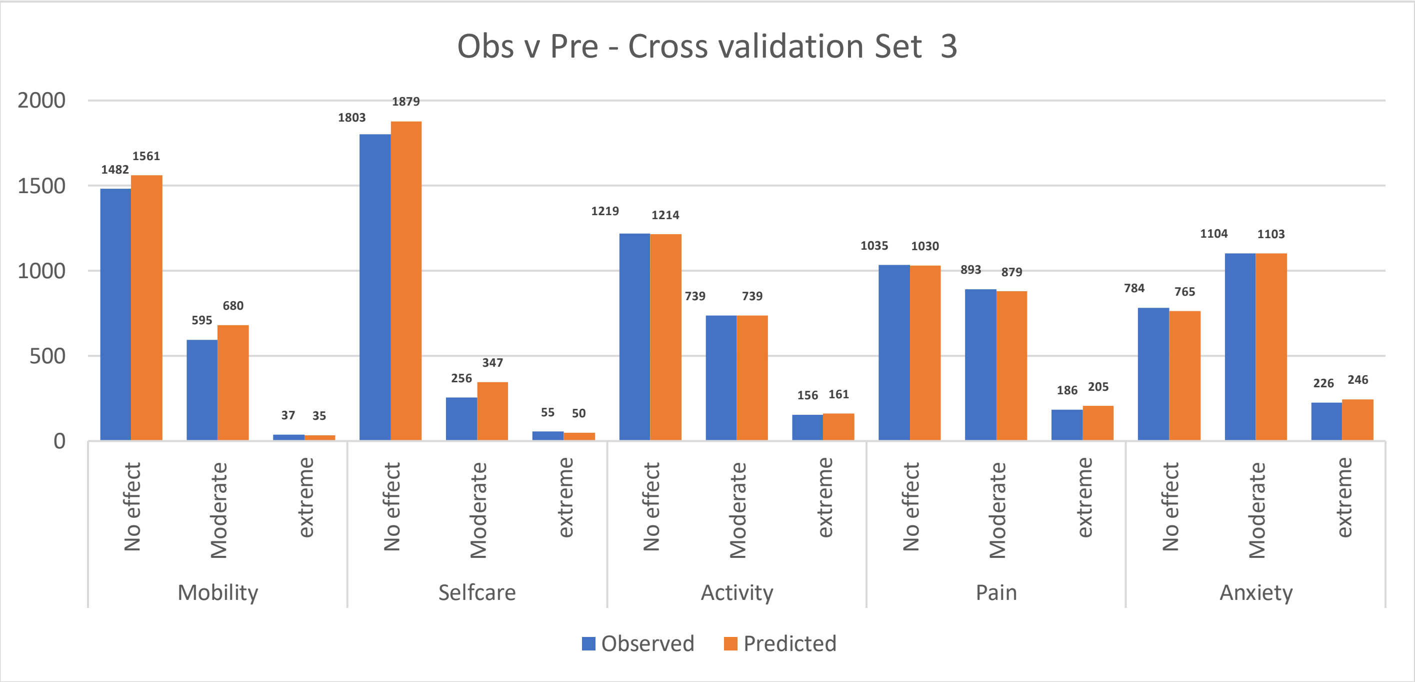
Obs v Pre - Cross validation Set 3
2000
1500
1000
500
0
1482 1561
595 680
37 35
1803 1879
256 347
55 50
1219 1214
739 739
156 161
1035 1030
893 879
186 205
784 765
1104 1103
226 246
No effect
Moderate
extreme
Mobility
Selfcare
Activity
Pain
Anxiety
Observed
Predicted

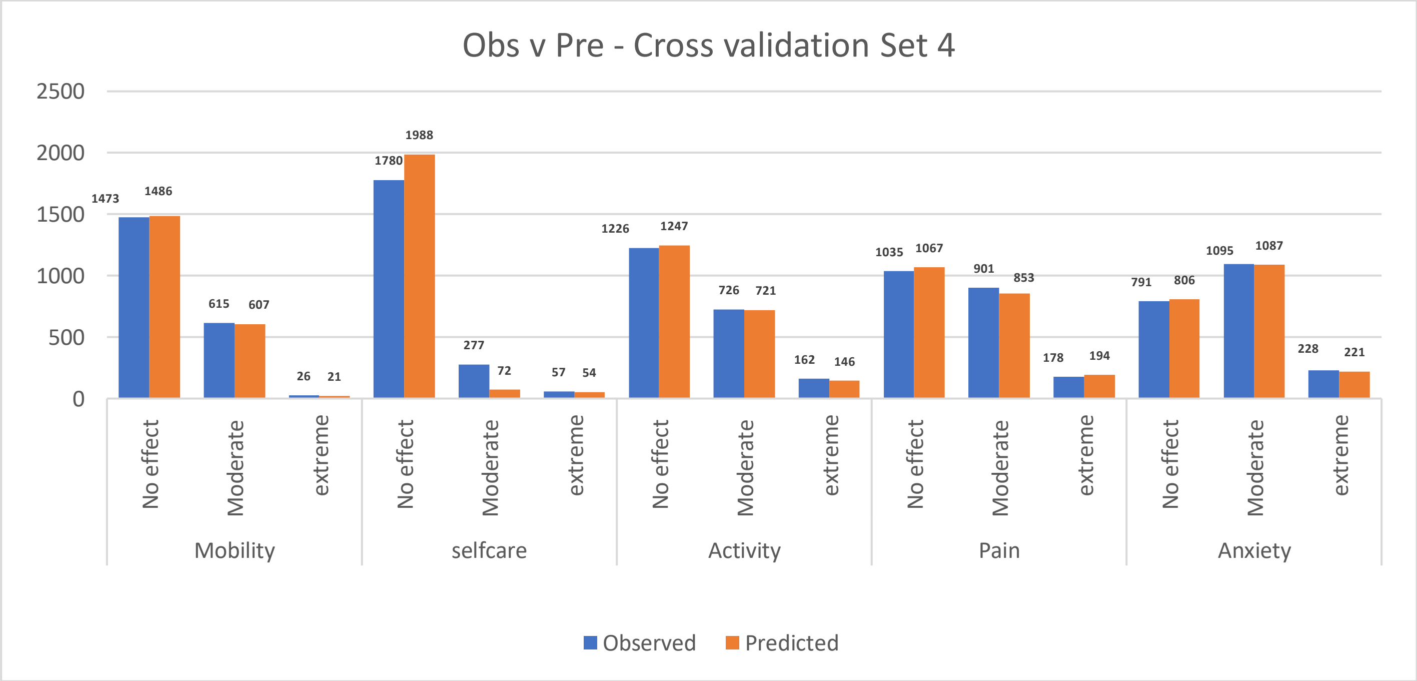
Obs v Pre - Cross validation Set 4
2500
2000
1500
1000
500
0
1473 1486
615 607
26 21
1780 1988
277 72
57 54
1226 1247
726 721
162 146
1035 1067
901 853
178 194
791 806
1095 1087
228 221
No effect
Moderate
extreme
Mobility
selfcare
Activity
Pain
Anxiety
Observed
Predicted

Fig S1 (Contd)

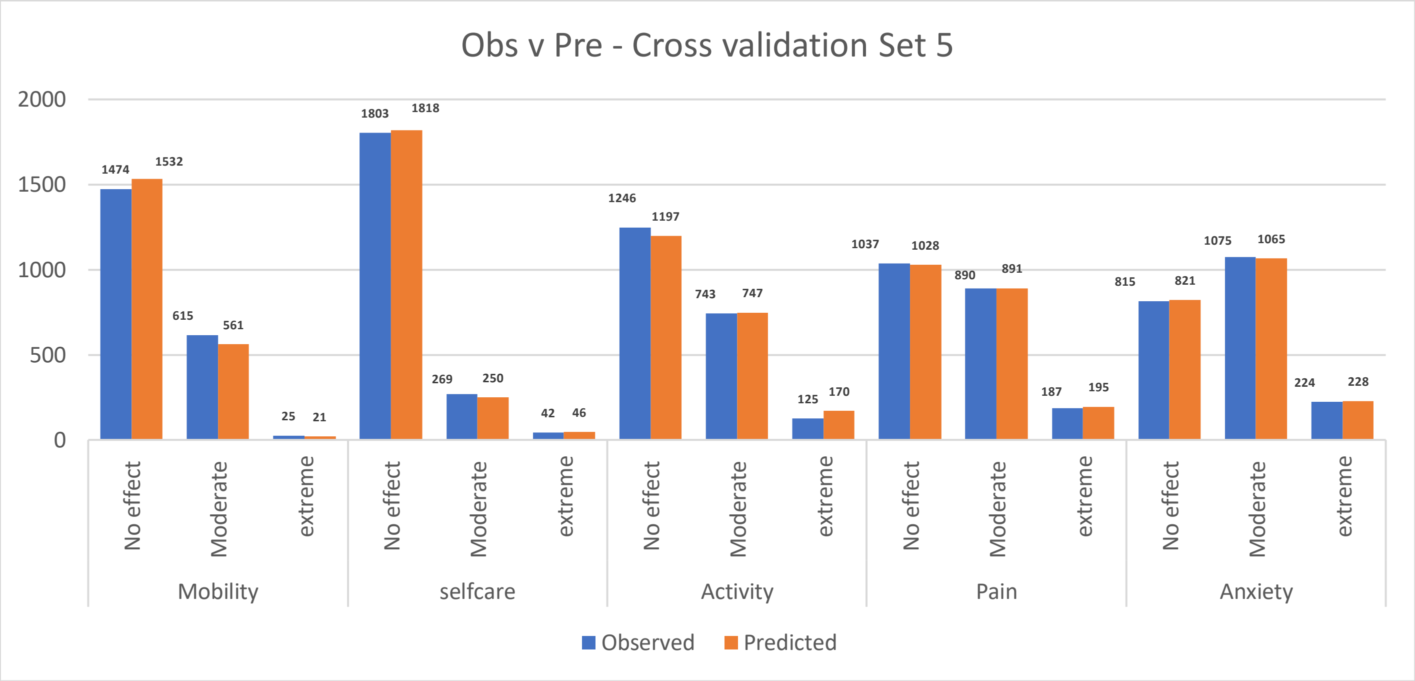
Obs v Pre - Cross validation Set 5
2000
1500
1000
500
0
1474
1532
615
561
25
21
1803
1818
269
250
42
46
1246
1197
743
747
125
170
1037
1028
890
891
187
195
815
821
1075
1065
224
228
No effect
Moderate
extreme
Mobility
selfcare
Activity
Pain
Anxiety
Observed
Predicted

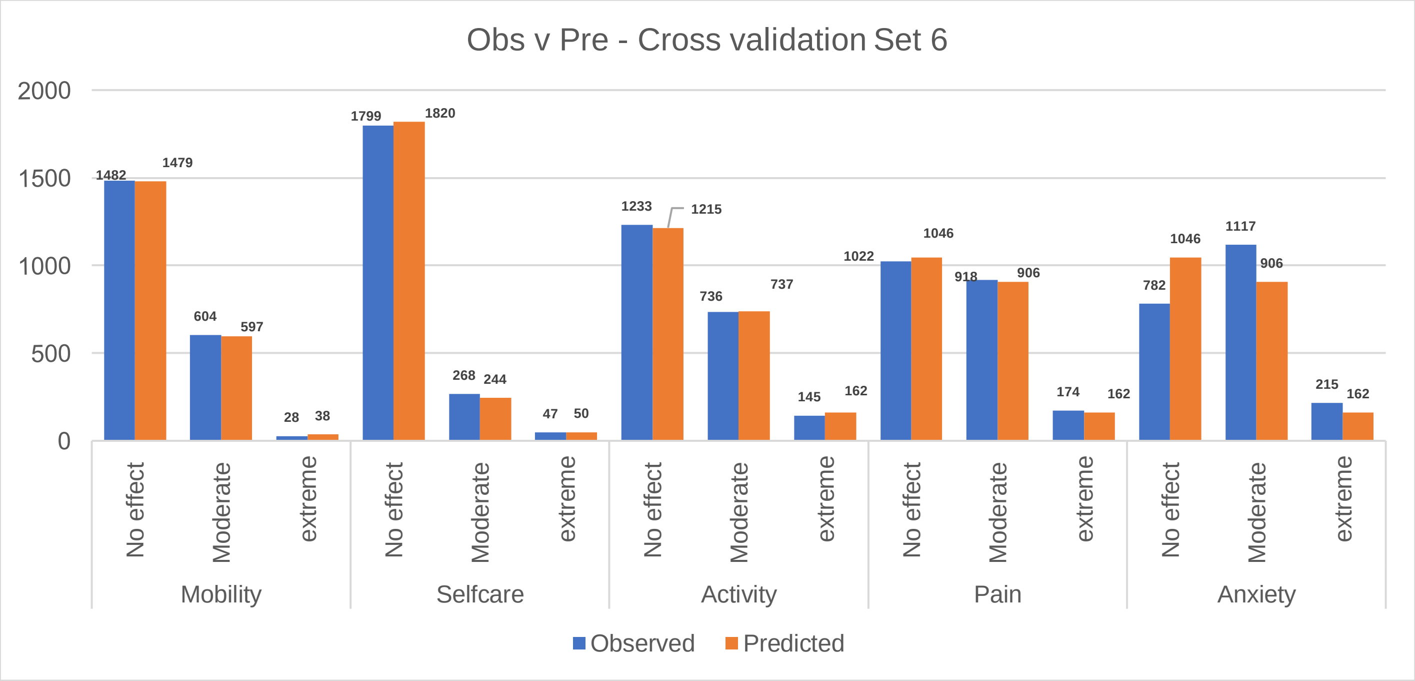
Obs v Pre - Cross validation Set 6
2000
1500
1000
500
0
1482
1479
604
597
28
38
1799
1820
268
244
47
50
1233
1215
736
737
145
162
1022
1046
918
906
174
162
782
1046
1117
906
215
162
No effect
Moderate
extreme
Mobility
Selfcare
Activity
Pain
Anxiety
Observed
Predicted

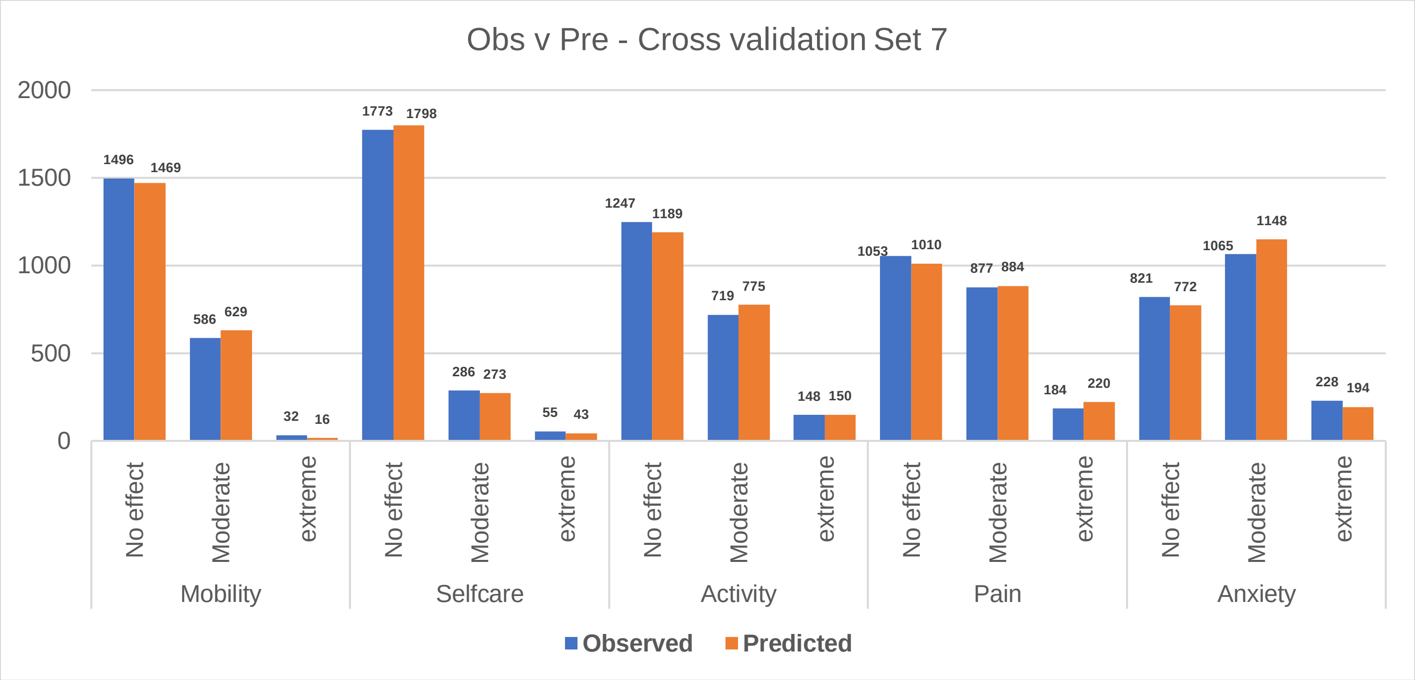
Obs v Pre - Cross validation Set 7
2000
1500
1000
500
0
1496
1469
586
629
32
16
1773
1798
286
273
55
43
1247
1189
719
775
148
150
1053
1010
877
884
184
220
821
772
1065
1148
228
194
No effect
Moderate
extreme
Mobility
Selfcare
Activity
Pain
Anxiety
Observed
Predicted

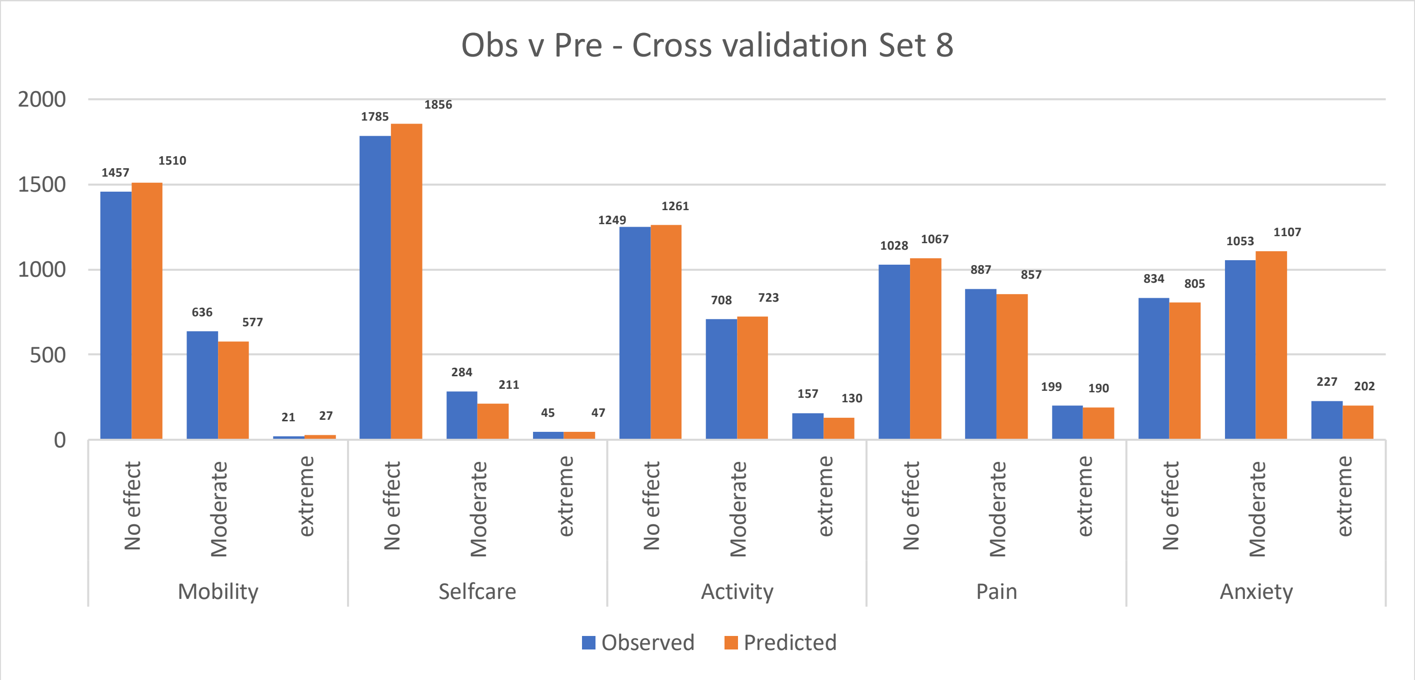
Obs v Pre - Cross validation Set 8
2000
1500
1000
500
0
1457
1510
636
577
21
27
1785
1856
284
211
45
47
1249
1261
708
723
157
130
1028
1067
887
857
199
190
834
805
1053
1107
227
202
No effect
Moderate
extreme
Mobility
Selfcare
Activity
Pain
Anxiety
Observed
Predicted

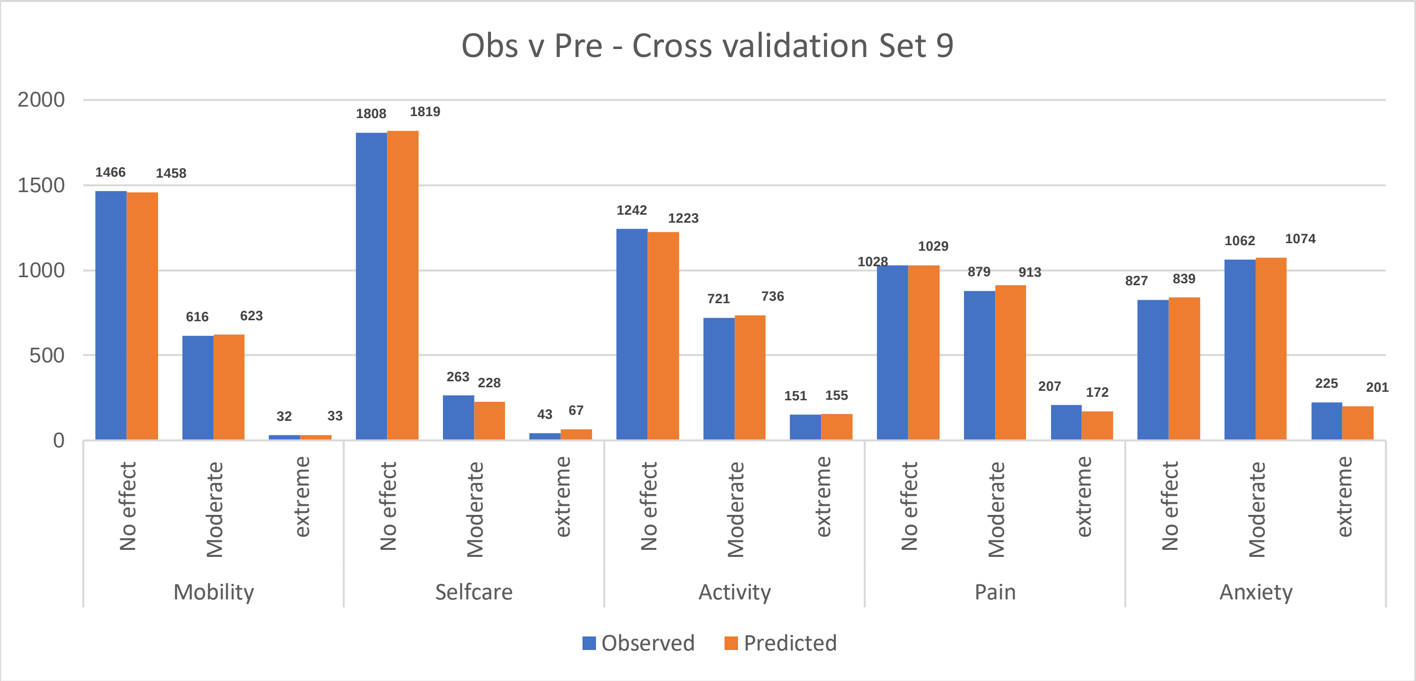
Obs v Pre - Cross validation Set 9
2000
1500
1000
500
0
1466
1458
616
623
32
33
1808
1819
263
228
43
67
1242
1223
721
736
151
155
1028
1029
879
913
207
172
827
839
1062
1074
225
201
No effect
Moderate
extreme
Mobility
Selfcare
Activity
Pain
Anxiety
Observed
Predicted

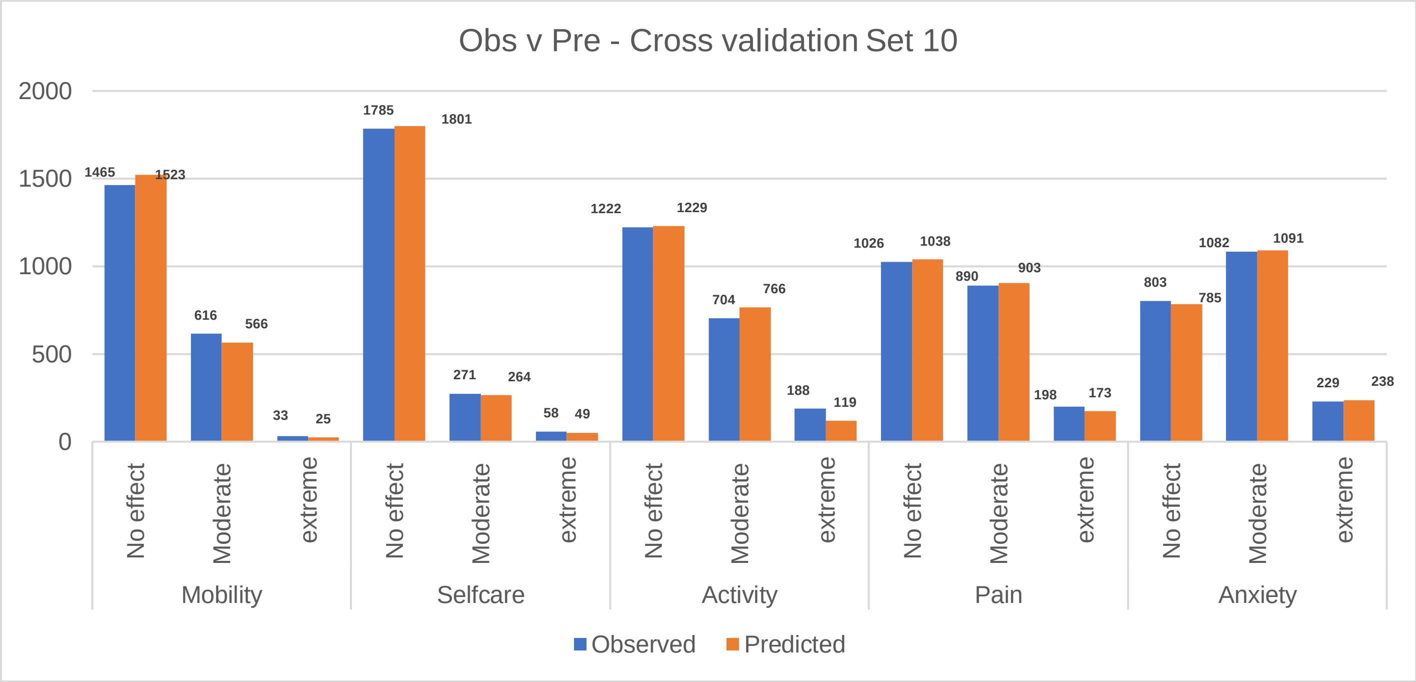
Obs v Pre - Cross validation Set 10
2000
1500
1000
500
0
1465
1523
616
566
33
25
1785
1801
271
264
58
49
1222
1229
704
766
188
119
1026
1038
890
903
198
173
803
785
1082
1091
229
238
No effect
Moderate
extreme
Mobility
Selfcare
Activity
Pain
Anxiety
Observed
Predicted

Fig S2 (a)-(j) The cumulative percentage of observed EQ-5D-3L data vs. simulated data across ten validation sets.

(a) Model one: Cross validation set 1

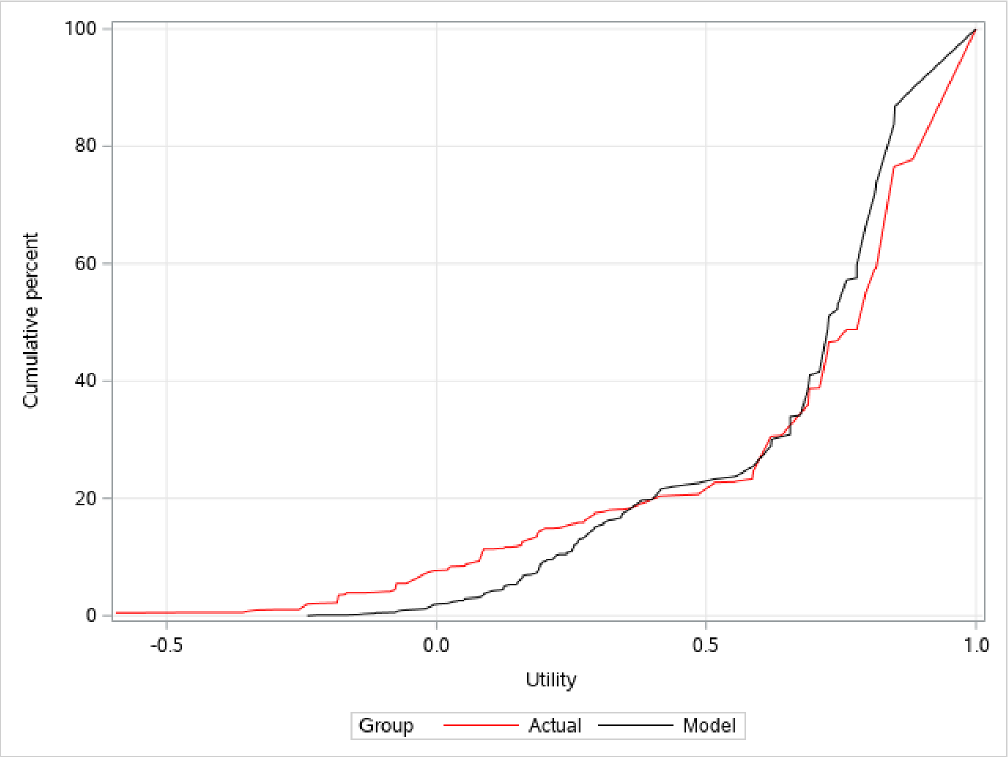

(b) Model one: Cross validation set 2

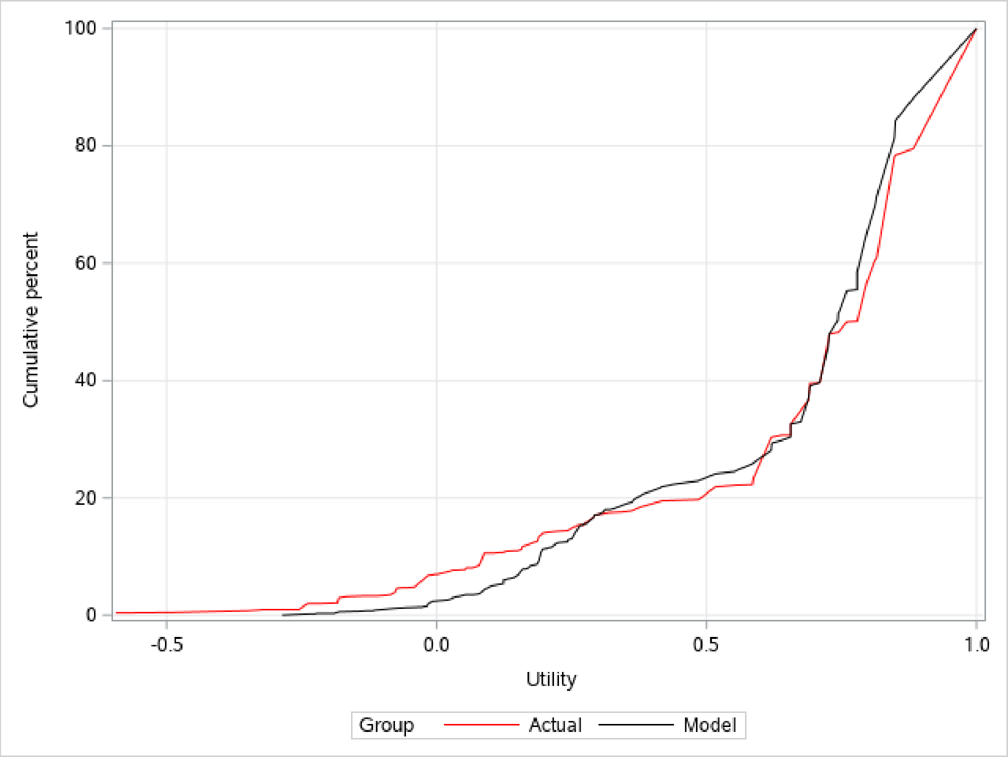

(c) Model three: Cross validation set 3

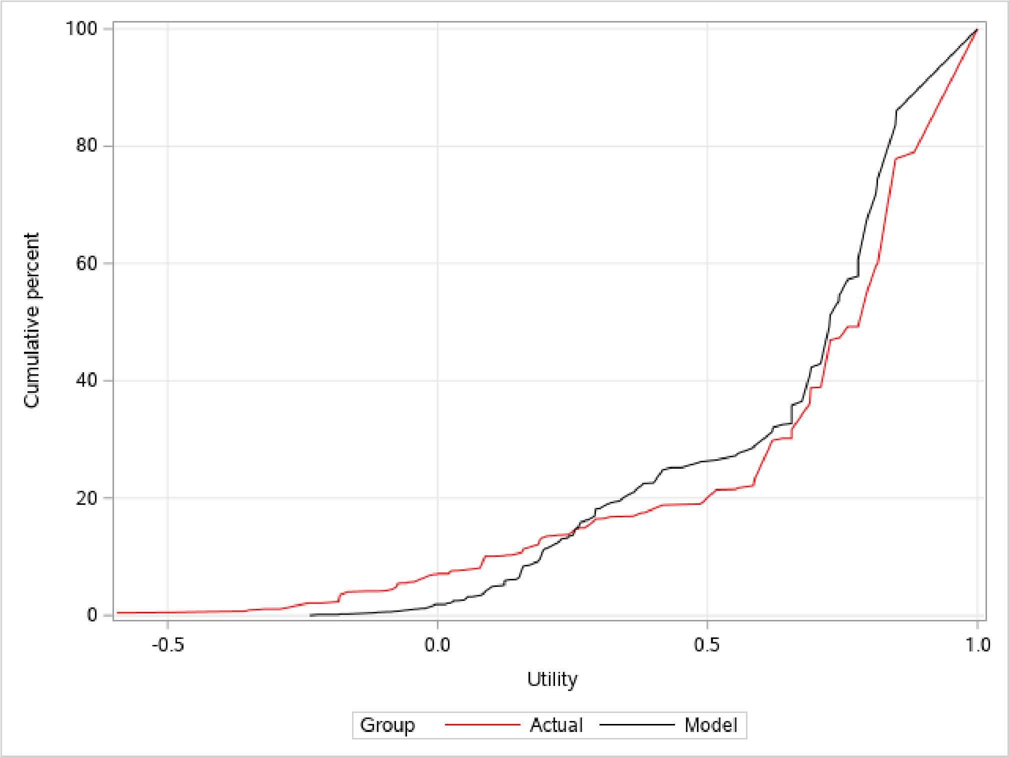

(d) Model four: Cross validation set 4

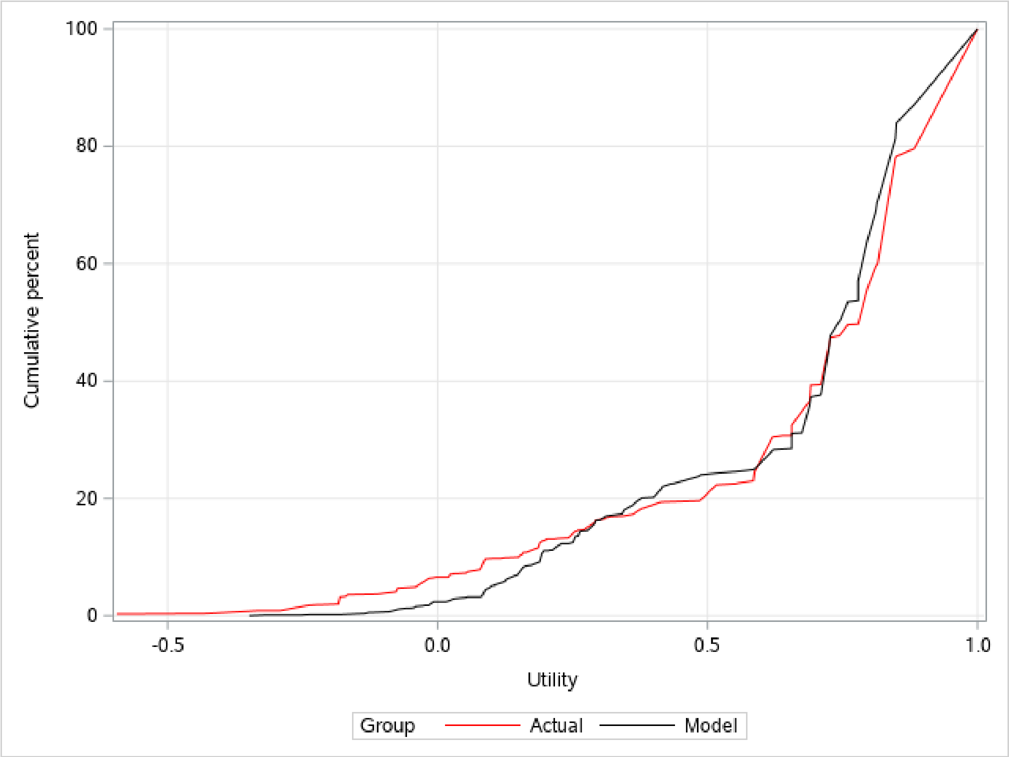

(e) Model five: Cross validation set 5

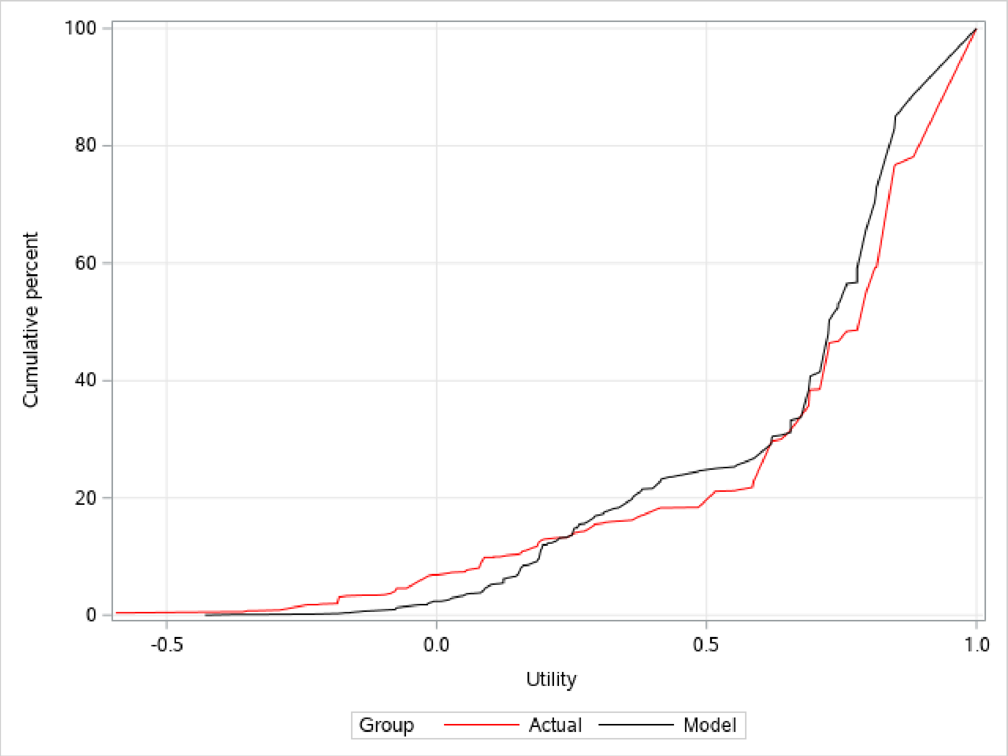

(f) Model six: Cross validation set 6

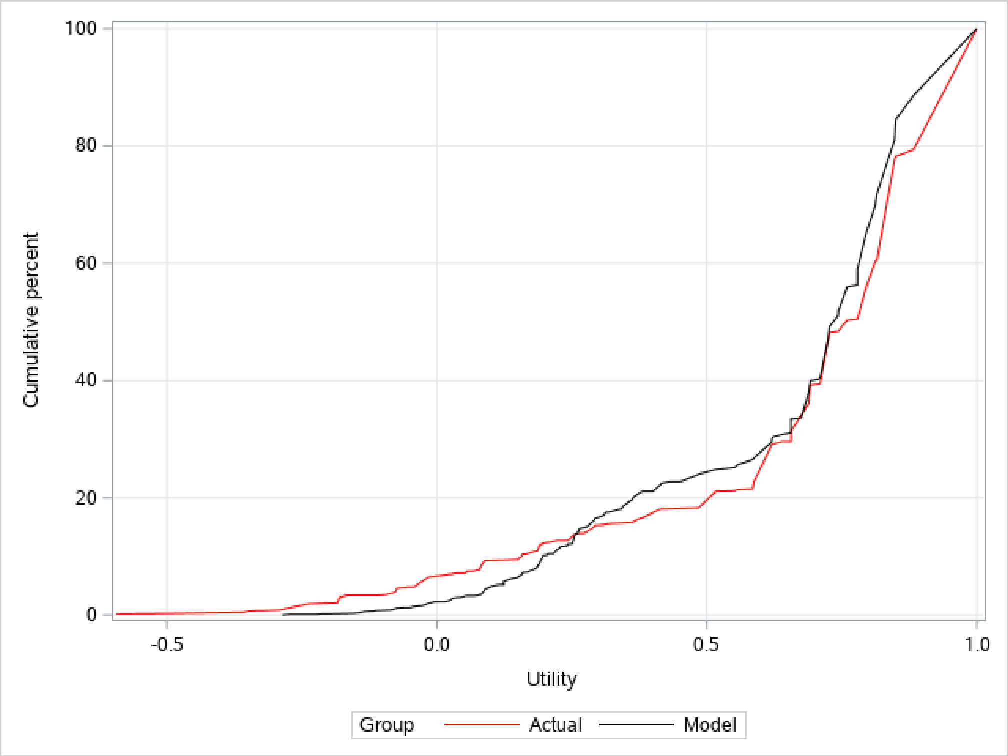

(g) Model seven: Cross validation set 7

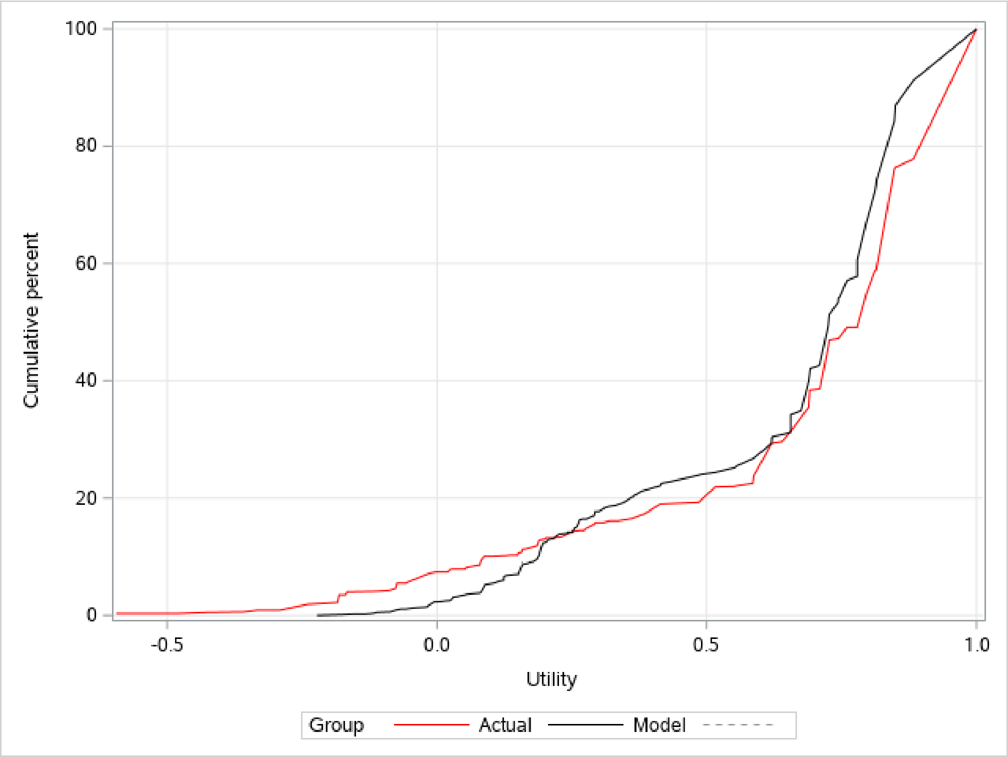

(h) Model eight: Cross validation set 8

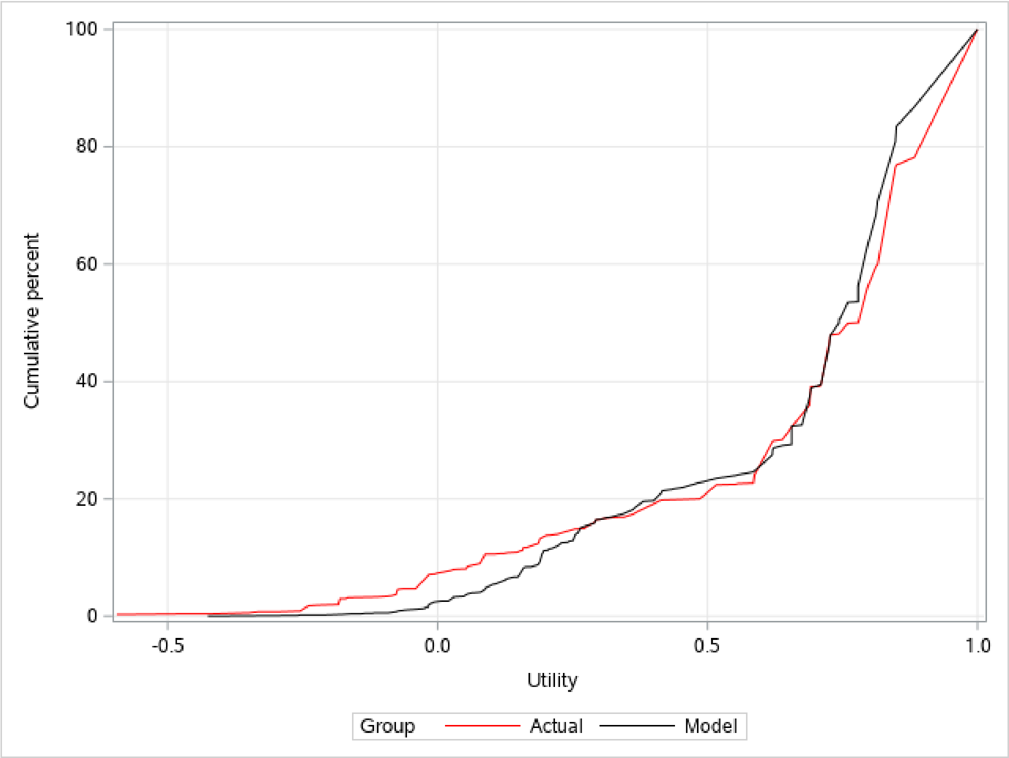

(i) Model Nine: Cross validation set 9

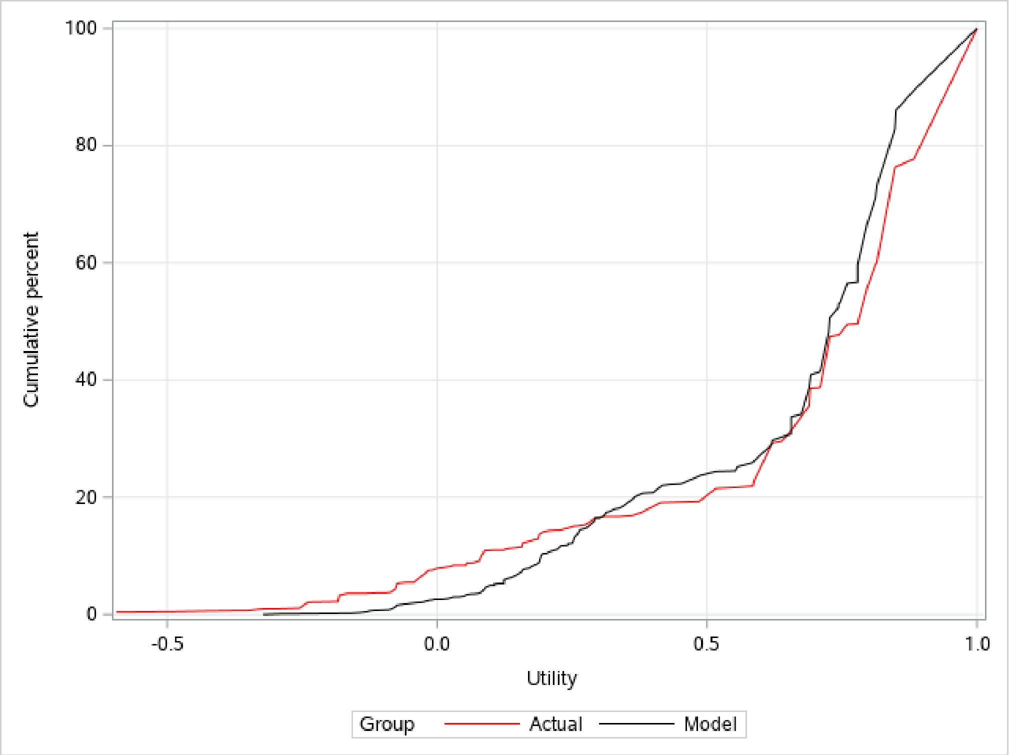

(j) Model ten: Cross validation set 10

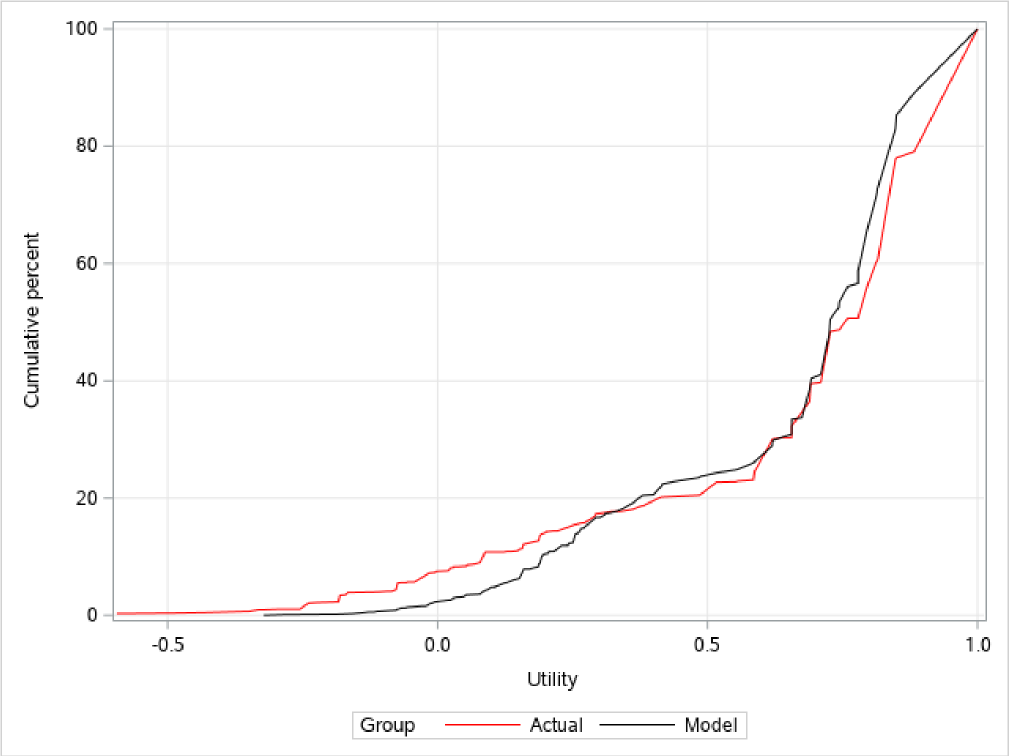

Fig S3 (a)-(j) Histograms demonstrating the mean difference between predicted and actual utility scores for each Monte Carlo simulation

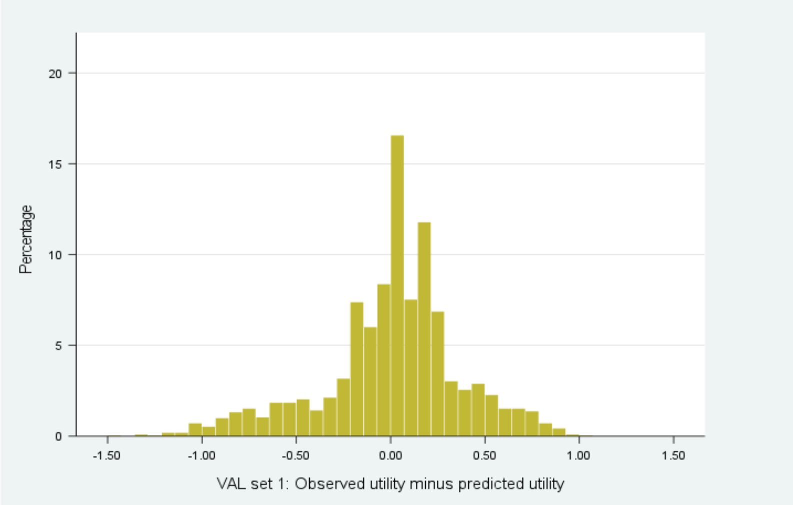

(a)

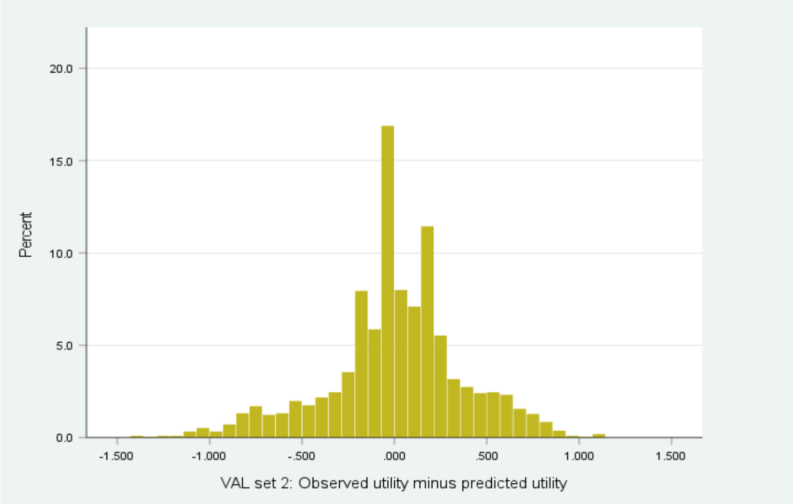

(b)

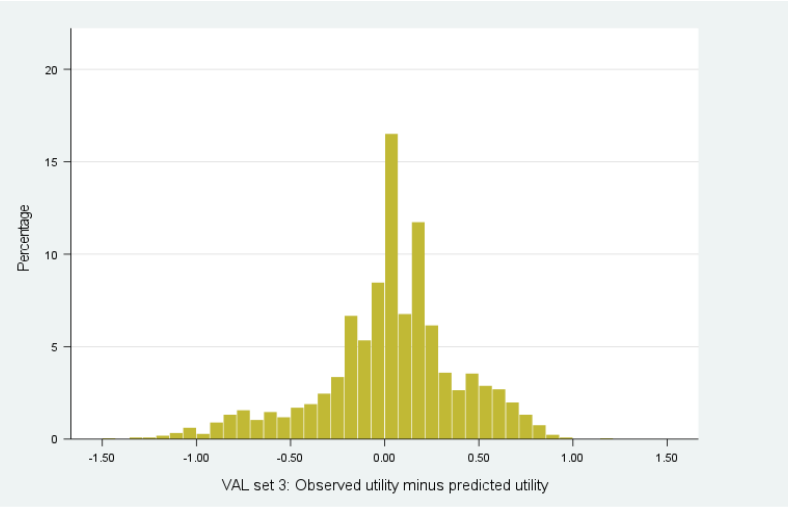

(c)

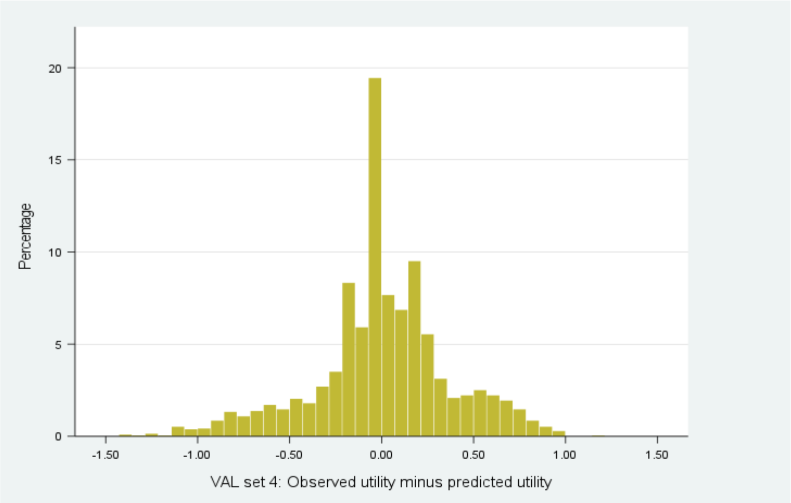

(d)

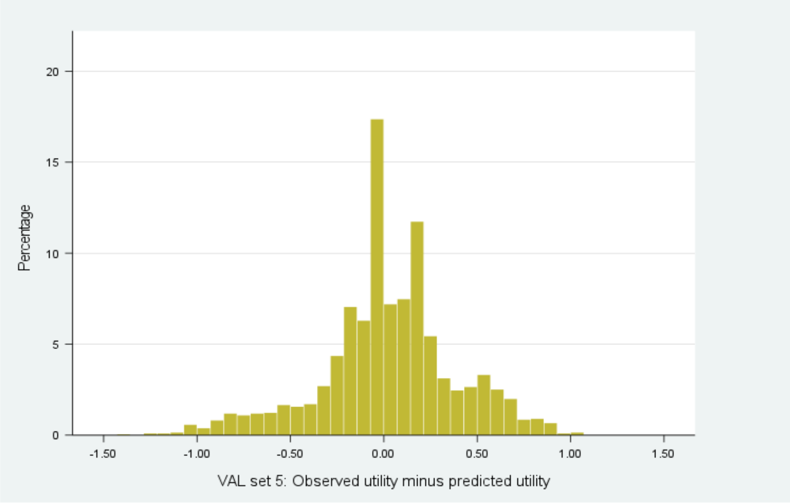

(e)

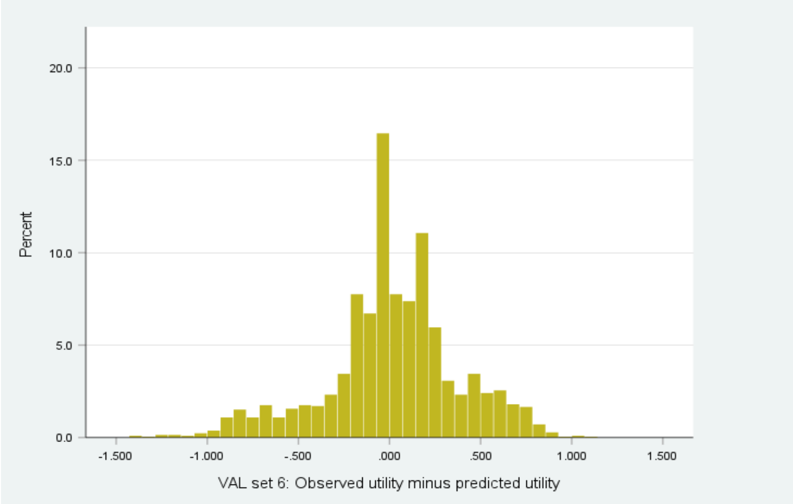

(f)

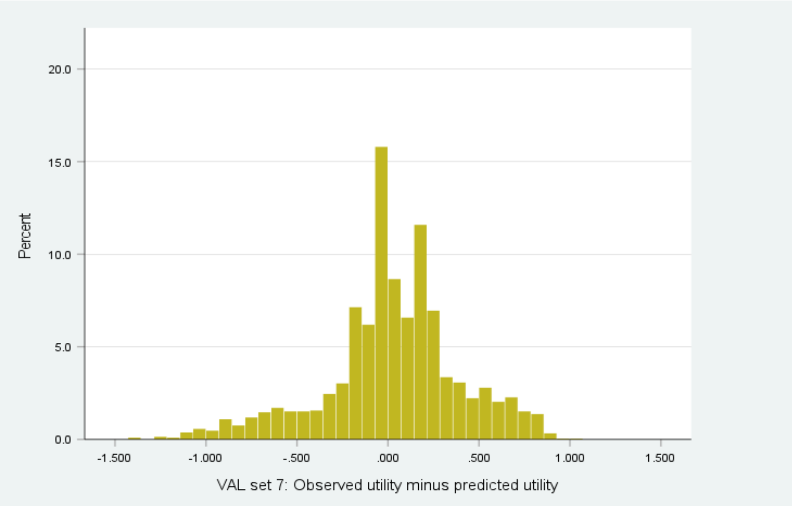
Percent
20.0
15.0
10.0
5.0
0.0
-1.500
-1.000
-.500
.000
.500
1.000
1.500
VAL set 7: Observed utility minus predicted utility

(g)

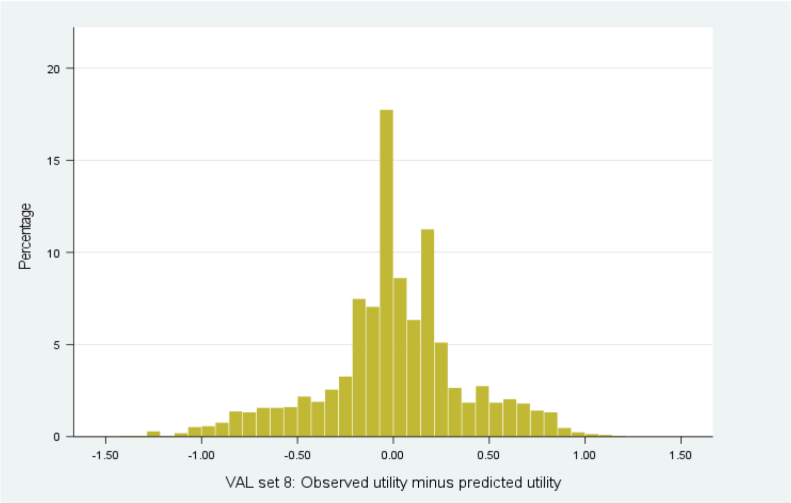
Percentage
20
15
10
5
0
-1.50
-1.00
-0.50
0.00
0.50
1.00
1.50
VAL set 8: Observed utility minus predicted utility

(h)

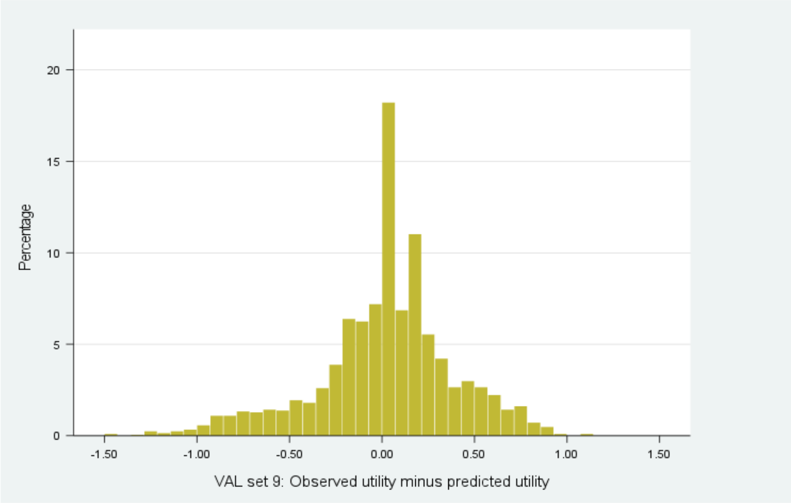
Percentage
20
15
10
5
0
-1.50
-1.00
-0.50
0.00
0.50
1.00
1.50
VAL set 9: Observed utility minus predicted utility

(i)

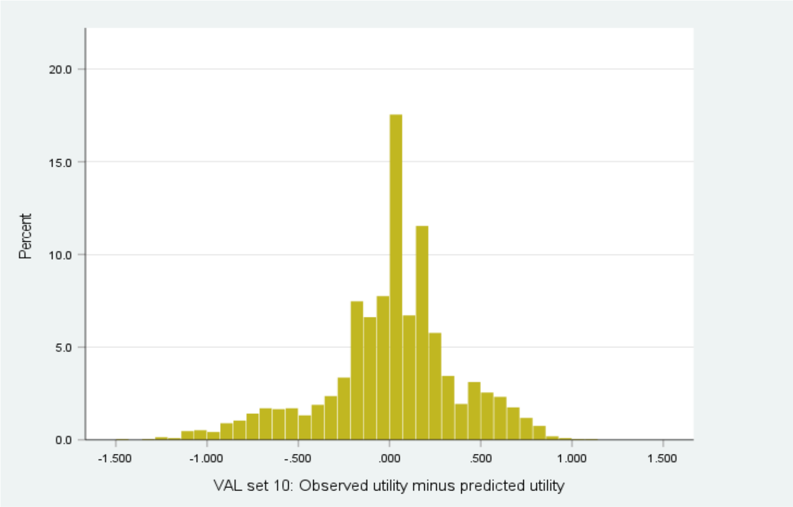
Percent
20.0
15.0
10.0
5.0
0.0
-1.500
-1.000
-.500
.000
.500
1.000
1.500
VAL set 10: Observed utility minus predicted utility

(j)
