# Supplementary material for: Mapping of Family Reported Outcome Measure (FROM-16) scores to EQ-5D: algorithm to calculate utility values
Source: Qual Life Res. 2024 Feb 25;33(4):1107–19. doi: 10.1007/s11136-023-03590-z (PMC10973087; doi:10.1007/s11136-023-03590-z)
Supplement: Supplementary file 2 — Supplementary file2 (PDF 395 KB) [file 11136_2023_3590_MOESM2_ESM.pdf]

Supplementary File 2

Mapping of Family Reported Outcome Measure (FROM-16) scores to EQ-5D: Algorithm to calculate Utility Values

Shah R, Salek MS, Finlay AY, Kay R, Nixon SJ, Otwombe K, Ali MF, Ingram JR

How to use the FROM-16 mapping algorithm

Corresponding author:

ShahR45@cardiff.ac.uk

Microsoft Excel spreadsheet© with pre-programmed formulae is available upon request

PAGE 1 of EXCEL Sheet -Mapping Algorithm

The mapping of the FROM-16 scores to utility values for use in economic appraisal should be based on aggregated group scores. The example provided is for illustrative purposes only and should not be replicated as such.

Step 1: Enter the respondent's age, sex, and the responses to the 16 items of FROM-16, as shown below in the Excel sheet

	Conversion of FROM-16 Item Score to EQ-5D utility values Using Multinomial Logistic Regression (Enter family member (FM) Age, Sex & FROM-16 scores below per subject)																		
	A	B	C	D	E	F	G	H	I	J	K	L	M	N	O	P	Q	R	S
**2**	**Subject ID**	**FM Age (years)**	**FM Sex M= 0, F=1**	**Worry**	**Angry**	**Sad**	**Frustrated**	**Talking about thoughts**	**Difficulty caring**	**Time for self**	**Travel**	**Eating habits**	**Family activities**	**Holiday**	**Sex life**	**Work or study**	**Family relationships**	**Family expenses**	**Sleep**
3																			
4	1	73	0	1	0	0	1	1	2	0	0	1	0	0	0	0	0	0	1
5																			
6																			

9
10
11
Step 2: Substitute the values of constant, regression coefficients and values of the independent variables from Table 5 in the manuscript in the two equations below (when using an Excel sheet, add values for each cell See the example on the next page: for example for "FM Age=73" we can simply write "B4").

12

$$\log\left(\frac{\Pi_2}{\Pi_1}\right) = \alpha_{mobility\ (Some\ problem)} + (\beta_{age} * Age) + (\beta_{sex} * Sex) + (\beta_{worry} * Worry) + (\beta_{angry} * Angry) + (\beta_{sad} * Sad) + (\beta_{frustrated} * Frustrated) + (\beta_{thoughts} * Talking\ about\ thoughts) + (\beta_{caring} * Difficulty\ caring) + (\beta_{time\ for\ self} * Time\ for\ self) + (\beta_{travel} * Tarvel) + (\beta_{eating\ habits} * Eating\ habits) + (\beta_{family\ activities} * Family\ activities) + (\beta_{holiday} * Holiday) + (\beta_{sex\ life} * Sex\ life) + (\beta_{work} * Work/study) + (\beta_{family\ realtionship} * Family\ realtionship) + (\beta_{family\ expenses} * Family\ expenses) + (\beta_{sleep} * Sleep)$$

$$\log\left(\frac{\Pi_3}{\Pi_1}\right) = \alpha_{mobility(Extrem\ problem)} + (\beta_{age} * Age) + (\beta_{sex} * Sex) + (\beta_{worry} * Worry) + (\beta_{angry} * Angry) + (\beta_{sad} * Sad) + (\beta_{frustrated} * Frustrated) + (\beta_{thoughts} * Talking\ about\ thoughts) + (\beta_{caring} * Difficulty\ caring) + (\beta_{time\ for\ self} * Time\ for\ self) + (\beta_{travel} * Tarvel) + (\beta_{eating\ habits} * Eating\ habits) + (\beta_{family\ activities} * Family\ activities) + (\beta_{holiday} * Holiday) + (\beta_{sex\ life} * Sex\ life) + (\beta_{work} * Work/study) + (\beta_{family\ realtionship} * Family\ realtionship) + (\beta_{family\ expenses} * Family\ expenses) + (\beta_{sleep} * Sleep)$$

α = the constant of the equation
β = the coefficient of the predictor or independent variables(here age, gender and 16 items of FROM-16), β is the effect of the independent variable on the log-odds of being in category 2(some problem) and category 3 (Extreme problem) instead of category 1(no problem) in above equations.

Microsoft Excel spreadsheet© with pre-programmed formulae is available upon request.

PAGE 2 of EXCEL Sheet -Mapping Algorithm using Multinomial logistic regression

Estimated probability of each score per EQ-5D domain per subject (copy formula* down each column up to the last subject)

1																									
2	U	V	W	X	Y	Z	AA	AB	AC	AD	AE	AF	AG	AH	AI	AJ	AK	AL	AM	AN	AO	AP	AQ	AR	AS
3	**MOBILITY**					**Selfcare**					**Usual activities**					**Pain/Discomfort**					**Anxiety/Depression**				
4	log(п2/п1)	log(п3/п1)	**P1**	**P2**	**P3**	log(п2/п1)	log(п3/п1)	**P1**	**P2**	**P3**	log(п2/п1)	log(п3/п1)	**P1**	**P2**	**P3**	log(п2/п1)	log(п3/п1)	**P1**	**P2**	**P3**	log(п2/п1)	log(п3/п1)	**P1**	**P2**	**P3**
5	-0.449	-5.984	0.609	0.389	0.002	-1.8200	-5.8340	0.858	0.139	0.003	-0.5460	-3.5690	0.622	0.360	0.018	0.0500	-1.3480	0.433	0.455	0.112	-0.8190	-3.1780	0.675	0.297	0.028
6																									
7																									
9																									
10																									

Using FROM -16 values from Table 1, Estimated probabilities can be calculated using probability equations derived from Multinomial logistic regression as below (Example of first row)

**Eq-5D-3L dimensions**	**Formula*** →		**P1**	**P2**	**P3**
**Mobility**	U=log(п2/п1)	-3.468+(B4*0.031)+(C4*-0.05)+(D4*0.276)+(E4*0.094)+(F4*-0.303)+(G4*-0.071)+(H4*0.199) +(I4*0.002)+(J4*-0.066)+ (K4*0.152)+ (L4*0.221)+(M4*0.083)+(N4*-0.004)+(O4*0.095)+(P4*-0.241)+(Q4*-0.072)+(R4*0.333)+(S4*0.127) = **-0.449**	1/(1+EXP(U4) +EXP(V4)) **= 0.609**	EXP(U4)/(1+EXP(U4) +EXP(V4)) **= 0.389**	=EXP(V4)/(1+EXP(U4) +EXP(V4)) **= 0.002**
	V=log(п3/п1)	-7.48+(B4*0.008)+(C4*-0.059)+(D4*-0.14)+(E4*0.288)+(F4*0.346)+(G4*- 0.078) +(H4*0.458)+(I4*0.341) +(J4*0.204) +(K4*0.018) +(L4*0.15)+(M4*0.211)+(N4*0.249)+(O4*-0.06)+(P4*-0.152)+(Q4*0.455)+(R4*0.479)+(S4*-0.16)= **-5.984**			
**Selfcare**	Z=log(п2/п1)	-3.25+(B4*0.004)+(C4*-0.297)+(D4*0.387)+(E4*0.009)+(F4*-0.136)+(G4*-0.024)+(H4*0.132) + (I4*0.064) +(J4*-0.159)+ (K4*0.187)+(L4*0.292)+(M4*0.031)+(N4*-0.08)+(O4*0.119)+(P4*-0.262)+(Q4*0.068) +(R4*0.346)+(S4*0.223) = **-1.8200**	1/(1+ EXP(Z4) +EXP(AA4)) **= 0.858**	=EXP(Z4)/(1+EXP (Z4)+EXP(AA4)) **= 0.139**	=EXP(AA4)/(1+EXP(Z4) +EXP(AA4)) **= 0.003**
	AA=log(п3/п1)	-6.497+(B4*0)+(C4*0.153)+(D4*-0.227)+(E4*0.221)+(F4*0.624)+(G4*-0.525)+(H4*0.172)+ (I4*0.68)+(J4*0.124)+ (K4*0.183)+(L4*0.141)+(M4*0.149)+(N4*0.275) +(O4*0)+(P4*0.227)+(Q4*0.081)+(R4*0.563) +(S4*-0.258) = **-5.8340**			
**Usual activities**	AE=log(п2/п1)	-2.746+(B4*0.019)+(C4*-0.089)+(D4*0.17)+(E4*0.042)+(F4*-0.211)+(G4*-0.042)+(H4*0.203) +(I4*0.09)+(J4*-0.038)+(K4*0.161)+(L4*0.107)+(M4*0.19)+(N4*0.027)+(O4*0.109)+(P4*0.037)+(Q4*-0.005)+(R4*0.3)+(S4*0.195) = **-0.5460**	1/(1+EXP(AE4) +EXP(AF4)) **= 0.622**	EXP(AE4)/(1+EXP (AE4)+EXP(AF4)) **= 0.360**	=EXP(AF4)/(1+EXP (AE4)+EXP(AF4)) **= 0.018**
	AF=log(п3/п1)	-5.344+(B4*0.009)+(C4*-0.205)+(D4*0.255)+(E4*0.137)+(F4*0.264)+(G4*-0.266)+(H4*0.192)+(I4*0.301)+(J4*0.228) +(K4*0.313)+(L4*0.202) +(M4*0.194)+(N4*0.083) +(O4*0.202) +(P4*0.032)+(Q4*0.044)+(R4*0.395)+(S4*0.133) =**-3.5690**			
**Pain/Discomfort**	AJ=log(п2/п1)	-2.18+(B4*0.026)+(C4*-0.008)+(D4*0.17)+(E4*0.081)+(F4*-0.328)+(G4*0.025)+(H4*0.134)+(I4*-0.121)+(J4*0.022) +(K4*0.093) +(L4*0.094)+(M4*0.092)+(N4*0.002)+(O4*0.116)+(P4*-0.133)+(Q4*0.076) +(R4*0.239)+(S4*0.151) = **0.0500**	1/(1+EXP(AJ4) +EXP(AK4)) **=0.433**	=EXP(AJ4)/(1+EXP (AJ4)+EXP(AK4)) **=0.455**	=EXP(AK4)/(1+EXP (AJ4)+EXP(AK4)) **=0.112**
	AK=log(п3/п1)	-4.437+(B4*0.017)+(C4*-0.247)+(D4*0.383)+(E4*0.129)+(F4*-0.352)+(G4*0.01)+(H4*0.323) +(I4*0.085)+(J4*-0.267) +(K4*0.305)+(L4*0.364)+(M4*0.035)+(N4*-0.027)+(O4*0.131)+(P4*-0.329)+(Q4*-0.063)+(R4*0.527)+(S4*0.598) = **-1.3480**			
**Anxiety/Depression**	AO=log(п2/п1)	-1.434+(B4*-0.013)+(C4*0.269)+(D4*0.522)+(E4*0.269)+(F4*0.117)+(G4*-0.021)+(H4*0.403)+(I4*0.029) +(J4*0.244) +(K4*0.102)+(L4*0.198)+(M4*0.1)+(N4*0.047)+(O4*0.069)+(P4*0.031)+(Q4*0.21)+(R4*-0.083)+(S4*0.404) = **-0.8190**	1/(1+EXP(AO4) +EXP(AP4)) **= 0.675**	EXP(AO4)/(1+EXP (AO4)+EXP(AP4)) **= 0.297**	EXP(AP4)/(1+EXP (AO4)+EXP(AP4)) **= 0.028**
	AP=log(п3/п1)	-4.012+(B4*-0.032)+(C4*-0.037)+(D4*0.715)+(E4*0.395)+(F4*0.353)+(G4*-0.085)+(H4*0.777)+(I4*0.223)+(J4*0.356) +(K4*0.154)+(L4*0.613)+(M4*0.059)+(N4*-0.214)+(O4*0.073)+(P4*-0.028)+(Q4*0.277)+(R4*0.082)+(S4*0.704) = **-3.1780**			

Microsoft Excel spreadsheet© with pre-programmed formulae is available upon request

PAGE 3 of EXCEL Sheet -Mapping Algorithm Monte Carlo Simulation method

	AU	AV	AW	AX	AY	AZ	BA	BB	BC	BD	BE	BF	BG
1	**Random Number Generator* for each domain** (formula* random number generator is given in the box below)					Monte Carlo Simulation	**Predicted score for each EQ-5D domain#** (formula for each dimension is given in the box below)					Conversion of EQ-5D-3L domain score to Utility value	**RESULTS**
2	**Mobility Random**	**Selfcare Random**	**Usual activities Random**	**Pain/Discomfort Random**	**Anxiety/Depression Random**		**Mobility Score**	**Selfcare Score**	**Usual Activities Score**	**Pain/ Discomfort Score**	**Anxiety/ Depression Score**		**Utility value #**
3													
4	0.4327	0.3375	0.4336	0.6160	0.7480		1	1	1	2	2		0.725
5													

The predicted domain scores may be converted to utility scores using the relevant TTO value sets specific to your country. These value sets as well as the syntax for SPSS (incase of large datasets) may be obtained from http://www.euroqol.org

#The utility value is calculated here using the UK MVH tariff equation developed by Dolan et al. 1996.

Step 3: Generate random numbers between 0 and 1 using the formula*=RAND()

Step 4: Predict the EQ-5D-3L domain response score using the formula below (linked to predicted domain probabilities on page 2)

Mobility score = IF(AU4=<W4,1,0)+IF(AND(AU4>W4,AU4=<(W4+X4)),2,0)+IF(AU4>(1-Y4),3,0)
Selfcare = IF(AV4=<AB4,1,0)+IF(AND(AV4>AB4,AV4=<(AB4+AC4)),2,0)+IF(AV4>(1-AD4),3,0)
Usual activities = IF(AW4=<AG4,1,0)+IF(AND(AW4>AG4,AW4=<(AG4+AH4)),2,0)+IF(AW4>(1-AI4),3,0)
Pain/Discomfort = IF(AX4=<AL4,1,0)+IF(AND(AX4=AL4,AX4=<(AL4+AM4)),2,0)+IF(AX4>(1-AN4),3,0)
Anxiety/Depression = =IF(AY4=<AQ4,1,0)+IF(AND(AY4>AQ4,AY4=<(AQ4+AR4)),2,0)+IF(AY4>(1-AS4),3,0)

Step 5: Utility value can now be calculated using UK TTO tariff. In this example we have calculated a health state of 11122 using the respondent's FROM-16 score, age and sex, which is equal to a utility value of 0.725. *Please note the algorithm is more accurate at the group level. The example shown here, with data from a single subject, is to inform readers how to use the mapping algorithm.

Microsoft Excel spreadsheet© with pre-programmed formulae is available upon request
